# A Polar Tetragonal Tungsten Bronze with Colossal Second‐Harmonic Generation

**DOI:** 10.1002/advs.202301374

**Published:** 2023-04-23

**Authors:** Yunseung Kuk, Seong Bin Bae, Sang Mo Yang, Kang Min Ok

**Affiliations:** ^1^ Department of Chemistry Sogang University Seoul 04107 Republic of Korea; ^2^ Department of Physics Sogang University Seoul 04107 Republic of Korea

**Keywords:** dipole moments, phase transitions, polarizable cations, second harmonic generation, tetragonal tungsten bronze, vacancy‐driven structural distortions

## Abstract

A polar tetragonal tungsten bronze, Pb_1.91_K_3.22_□_0.85_Li_2.96_Nb_10_O_30_ (□: vacancies), has been successfully synthesized by a high temperature solid‐state reaction. Single crystal and powder X‐ray diffraction indicate that the structure of Pb_1.91_K_3.22_□_0.85_Li_2.96_Nb_10_O_30_ crystallizing in the noncentrosymmetric (NCS) space group, *P*4*bm*, consists of 3D framework with highly distorted NbO_6_, LiO_9_, PbO_12_, and (Pb/K)O_15_ polyhedra. While NCS Pb_1.91_K_3.22_□_0.85_Li_2.96_Nb_10_O_30_ undergoes a reversible phase transition between polar (*P*4*bm*) and nonpolar (*P*4/*mbm*) structure at around 460 °C, the material decomposes to centrosymmetric Pb_1.45_K_3.56_Li_3.54_Nb_10_O_30_ (*P*4/*mbm*) once heated to 1200 °C. Powder second‐harmonic generation (SHG) measurements with 1064 nm radiation indicate that Pb_1.91_K_3.22_□_0.85_Li_2.96_Nb_10_O_30_ exhibits a giant phase‐matchable SHG intensity of ≈71.5 times that of KH_2_PO_4_, which is the strongest intensity in the visible range among all nonlinear optical materials reported to date. The observed colossal SHG should be attributable to the synergistic effect of dipole moments from the well‐aligned NbO_6_ octahedra, the constituting distortive channels with vacancies, and highly polarizable cations.

## Graphical abstract

A polar tungsten bronze, Pb_1.91_K_3.22_□_0.85_Li_2.96_Nb_10_O_30_, reveals extremely large second‐harmonic generation efficiency of ≈71.5 times that of KH_2_PO_4_ attributed to the constructive effect of moments arising from the well‐aligned distorted NbO_6_ octahedra, structural distortions at the quadrangular sites with vacancies, and strong interactions of the backbone with highly polarizable cations.

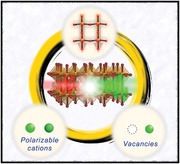

## 1 Introduction

Discovering novel materials with macroscopic noncentrosymmetric (NCS) structures has drawn tremendous attention owing to their unique structure‐related characteristics such as ferroelectricity, piezoelectricity, pyroelectricity, and nonlinear optical (NLO) properties.^[^ ^1^ ^]^ Among them, second‐harmonic generation (SHG), that is, one of the second‐order NLO properties, can effectively generate a new frequency doubled coherent light from the fundamental light, which is a pivotal property in a variety of advanced industries.^[^ ^2^ ^]^ Although a number of superb NLO materials such as LiNbO_3_, KH_2_PO_4_ (KDP), KTiOPO_4_, etc. have been developed to date,^[^ ^3^ ^]^ discovering novel functional materials with strong SHG response and wide transparency window is still an ongoing challenge. Thus far, introducing NCS chromophores with large polarization, such as two families of cations susceptible to second‐order Jahn‐teller (SOJT) distortions, for example, d^0^ transition metal cations in the octahedral coordination environments (Ti^4+^, V^5+^, Nb^5+^, Mo^6+^) and stereochemically active lone pair cations (Pb^2+^, Sb^3+^, Bi^3+^, etc.), and *π*‐delocalized anionic groups (NO_3_^−^, CO_3_^2−^, BO_3_^3−^) have been widely utilized during the synthesis to ensure high‐performance NLO materials.^[^ ^4^ ^]^ Unfortunately, however, the majority of the reaction products are still found to be thermodynamically stable centrosymmetric (CS) structures owing to the facile alignment of the local asymmetric units in an antiparallel manner. Therefore, exploring suitable frameworks with well‐aligned building units along a specific direction is extremely important.

Rigid frameworks of tungsten bronzes (TBs) consist of corner‐sharing MO_6_ (M = Ti^4+^, V^5+^, Nb^5+^, etc.) octahedra, from which various cations and vacancies are observed in the constituting channels. In particular, since the structural distortion of TB can be enhanced from the distorted MO_6_ octahedra as well as the polarizable cations and vacancies in the channels, the material has a perfect structural feature as a terrific NLO material. In fact, a few polar TB materials such as Pb_2.15_(Li_0.25_Na_0.75_)_0.7_Nb_5_O_15_ (47 × KDP; *Bb*2_1_ *m*), Pb_2.15_Li_0.6_Nb_5_O_15_ (44 × KDP; *Pn*2_1_ *m*), Pb_2_Li_0.94_RE_0.02_Nb_5_O_15_ (RE = Eu and Gd; 41 × KDP; *Pn*2_1_ *m*), Pb_2_(Pb_0.15_Li_0.7_□_0.15_)Nb_5_O_15_ (39 × KDP; *Pn*2_1_ *m*), Pb_2_KNb_5_O_15_ (25 × KDP; *Cm*2*m*), Pb_2_AgNb_5_O_15_ (100 × *α*‐SiO_2_; *Cm*2*m*), PbBiNb_5_O_15_ (0.3 × KDP; *Cm*2*m*), etc., exhibit very strong SHG responses.^[^ ^5^ ^]^ Previously reported TB with distorted NbO_6_ octahedra and polarizable cations, Pb_0.91_K_1.72_Li_1.46_Nb_5_O_15_, might be also expected to exhibit a strong SHG response.^[^ ^6^ ^]^ In this work, we have successfully synthesized a novel polar tetragonal tungsten bronze (TTB), Pb_1.91_K_3.22_□_0.85_Li_2.96_Nb_10_O_30_, via a high temperature solid‐state reaction. The reported TTB material reveals a remarkably strong SHG intensity of ≈71.5 times that of KDP, which is the strongest SHG response in the visible range among the reported NLO materials thus far. We believe that exploring TBs with proper chemical compositions may accelerate the development of novel NLO materials with extremely strong SHG efficiency.

## 2 Results and Discussions

**Figure 1:**
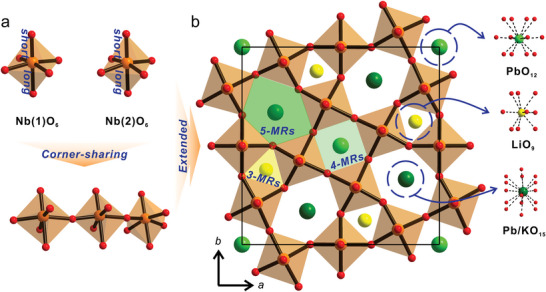
Ball‐and‐stick and polyhedral representations of Pb_1.91_K_3.22_□_0.85_Li_2.96_Nb_10_O_30_ revealing a) Nb(1)O_6_ and Nb(2)O_6_ octahedra with C_4_ octahedral distortions and b) extended structure in the *ab*–plane. Three types of polyhedra, that is, LiO_9_, PbO_12_, and (Pb/K)O_15_ reside at 3‐, 4‐, and 5‐MR channels, respectively (dark green, Pb/K; light green, Pb; yellow, Li; orange, Nb; red, O).

Single crystal X‐ray diffraction (SC‐XRD) indicates that Pb_1.91_K_3.22_□_0.85_Li_2.96_Nb_10_O_30_ crystallizing in the NCS polar tetragonal space group, *P*4*bm* (No. 100) reveals a 3D TTB structure containing two Nb, one disordered Pb/K, one partially occupied Pb, one partially occupied Li, and five O atoms in an asymmetric unit (Figure S1, Supporting Information). Two unique Nb^5+^ cations, Nb(1) and Nb(2) are connected by six oxygen atoms, forming NbO_6_ distortive octahedra with the Nb−O distances of 1.84(5)–2.20(5) Å (Table S2, Supporting Information). Both Nb(1)O_6_ and Nb(2)O_6_ octahedra exhibit one short, one long, and four intermediate Nb—O bonds in C_4_ distortive octahedral environments, in which the highly unsymmetrical NbO_6_ octahedral units are attributed to the SOJT effect (**Figure** **1**a). Each distorted Nb(1)O_6_ and Nb(2)O_6_ polyhedron shares its corners through oxygen atoms and constitutes a 3D framework structure containing 3‐ (3‐MR), 4‐ (4‐MR), and 5‐membered ring (5‐MR) channels (Figure 1a,b). While partially occupied Li^+^ cations reside in the 3‐MRs, disordered Pb^2+^/K^+^ cations occupy 5‐MR channels with the refined occupancies of 0.74(4) and 0.8057(13)/0.1943(13), respectively. The Li−O and Pb/K−O contact distances in LiO_9_ and (Pb/K)O_15_ polyhedra are 2.16(2)–2.62(17) Å and 2.709(8)–3.576(12) Å, respectively (Tables S2 and S3, Supporting Information). In addition, another partially occupied Pb^2+^ cations with the refined occupancy of 0.5711(12) are located at the quadrangular sites (4‐MRs) along with vacancies, in which the observed Pb−O lengths in PbO_12_ polyhedra are 2.613(9)–3.019(10) Å (Tables S2 and S3, Supporting Information). Thus, the overall structure of the reported compound can be classified as a TTB with a chemical formula of Pb_1.91_K_3.22_□_0.85_Li_2.96_Nb_10_O_30_. In fact, the crystallographically refined formula matches very well with those obtained from the energy dispersive X‐ray (EDX) analysis as well as inductively coupled plasma optical emission spectroscopy (ICP‐OES) (Figure S2, Supporting Information). Also, a sharp signal at the chemical shift of 1.10 ppm from the ^7^Li solid‐state magic angle spinning nuclear magnetic resonance (MAS NMR) spectrum of Pb_1.91_K_3.22_□_0.85_Li_2.96_Nb_10_O_30_ confirms the presence of Li in the 3‐MR channels (Figure S3, Supporting Information).

The infrared (IR) spectrum of Pb_1.91_K_3.22_□_0.85_Li_2.96_Nb_10_O_30_ reveals vibrations arising from the constituting polyhedra in the range of 400–1000 cm^−1^ (Figure S4, Supporting Information). While the vibrational band for Pb—O occurs at ≈520 cm^−1^, broad bands for Nb—O bonds are found at ≈455–850 cm^−1^. Peaks appearing at ≈400–440 cm^−1^ may be attributed to the Li—O and K—O interactions. The assignments are consistent with the previously reported compounds composed of niobium‐based frameworks.^[^ ^7^ ^]^

The optical band gap calculated by the Kubelka–Munk equation using the collected ultraviolet–visible (UV–vis) reflectance data suggests that Pb_1.91_K_3.22_□_0.85_Li_2.96_Nb_10_O_30_ has a wide band gap of 3.45 eV attributed to the presence of distorted NbO_6_ polyhedra (Figure S5, Supporting Information).^[^ ^8^ ^]^ A few Pb‐based TB structures with wide band gaps include Pb_2_AgNb_5_O_15_ (2.64 eV),^[^ ^5c^ ^]^ Pb_2.15_(Li*_x_* Na_1‐_ *_x_*)_0.7_Nb_5_O_15_ (0 ≤ *x* ≤ 1; 2.81−2.84 eV),^[^ ^5a^ ^]^ and Pb_2_(Pb_0.15_Li_0.7_□_0.15_)Nb_5_O_15_ (2.84 eV) (Table S5, Supporting Information).^[^ ^5b^ ^]^ To the best of our knowledge, the title compound has the largest band gap among the reported Pb‐based TBs.

**Figure 2:**
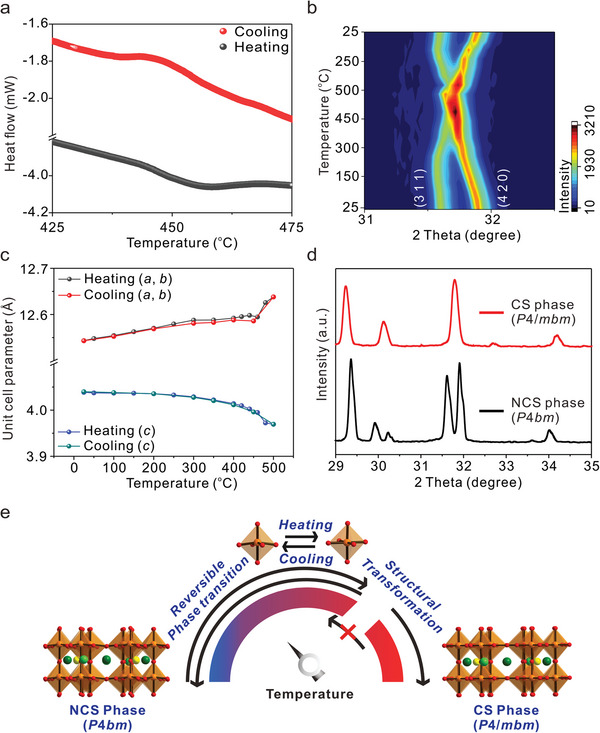
a) DSC curves in the temperature range of 425–475 °C, b) 2D contour plots of in situ PXRD patterns in the 2*θ* range of 31°–32.5°, and c) plots of unit cell parameters versus temperature for Pb_1.91_K_3.22_□_0.85_Li_2.96_Nb_10_O_30_. d) Magnified PXRD patterns in the 2*θ* range of 29°–35° for NCS Pb_1.91_K_3.22_□_0.85_Li_2.96_Nb_10_O_30_ and CS Pb_1.45_K_3.56_Li_3.54_Nb_10_O_30_. e) Schematic illustration revealing the phase transitions of Pb_1.91_K_3.22_□_0.85_Li_2.96_Nb_10_O_30_ at different temperatures.

The thermogravimetric analysis (TGA) diagram shows that Pb_1.91_K_3.22_□_0.85_Li_2.96_Nb_10_O_30_ does not reveal any weight loss up to 900 °C (Figure S6, Supporting Information). However, differential scanning calorimetry (DSC) and temperature‐dependent in situ PXRD suggest that the material exhibits a reversible phase transition at ≈460 °C (**Figure** **2**a,b, and Figure S7, Supporting Information). As seen in Figure 2b and Figure S7, Supporting Information, peaks at ≈2*θ* = 21.8°−22.5°, 31.5°−31.9°, and 44.8°−45.8° get closer and coalesce with increasing temperature. The change of unit cell parameters for phases measured at different temperatures has been more closely analyzed by the GSAS‐II program using the in situ PXRD data (Figure 2c). While the unit cell parameters for *a* and *b* gradually increase with temperature, those for *c* gradually decrease up to 450 °C. However, the unit cell parameters sharply change as soon as it passes the phase transition temperature, 460 °C. As seen in the PXRD patterns and final Rietveld refinement plots measured at 480 °C, the high‐temperature CS phase (*P*4/*mbm*) is clearly distinct from the NCS phase (*P*4*bm*) (Figures S8 and S9, Supporting Information). As described, the phase transition of Pb_1.91_K_3.22_□_0.85_Li_2.96_Nb_10_O_30_ occurs reversibly upon heating and cooling between RT and ≈500 °C. Interestingly, however, Pb_1.91_K_3.22_□_0.85_Li_2.96_Nb_10_O_30_ undergoes an irreversible phase transition once the polycrystalline sample of the title material is heated to 1200 °C (Figure 2d,e, and Figure S10, Supporting Information). SC‐XRD on a colorless rod‐shaped crystal obtained upon fast cooling from the high temperature indicates that the decomposed material is Pb_1.45_K_3.56_Li_3.54_Nb_10_O_30_ crystallizing in the tetragonal CS space group, *P*4/*mbm* (No. 127). Pb_1.45_K_3.56_Li_3.54_Nb_10_O_30_ is another class of TTB structure composed of NbO_6_, (Pb/K)O_15_, PbO_12_, and LiO_9_ polyhedra. However, unlike the polar NCS structure, the NbO_6_ octahedra in CS Pb_1.45_K_3.56_Li_3.54_Nb_10_O_30_ exhibit similar Nb−O distances ranging from 1.937(6) to 2.001(5) Å (Figure S11, Supporting Information). Besides, the small moments arising from the slightly distorted NbO_6_ octahedra effectively cancel in the CS structure when taken as a whole (Table S4, Supporting Information).

**Figure 3:**
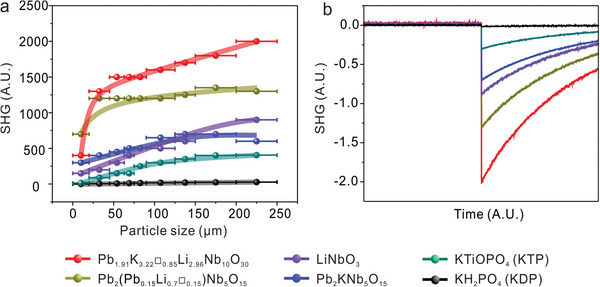
a) Plots of SHG intensity versus particle size and b) oscilloscope signals of the SHG responses in the particle size range of 200–250 µm for representative SHG materials.

Powder SHG measurements using 1064 nm radiation indicate that NCS Pb_1.91_K_3.22_□_0.85_Li_2.96_Nb_10_O_30_ exhibits an extremely large SHG intensity of about 71.5 times that of KDP and the type‐I phase‐matching behavior (**Figure** **3**a,b). It should be noticed that to the best of our knowledge, the measured SHG intensity of the title material is the strongest in the visible range among all the reported NLO materials (Table S5, Supporting Information).

**Figure 4:**
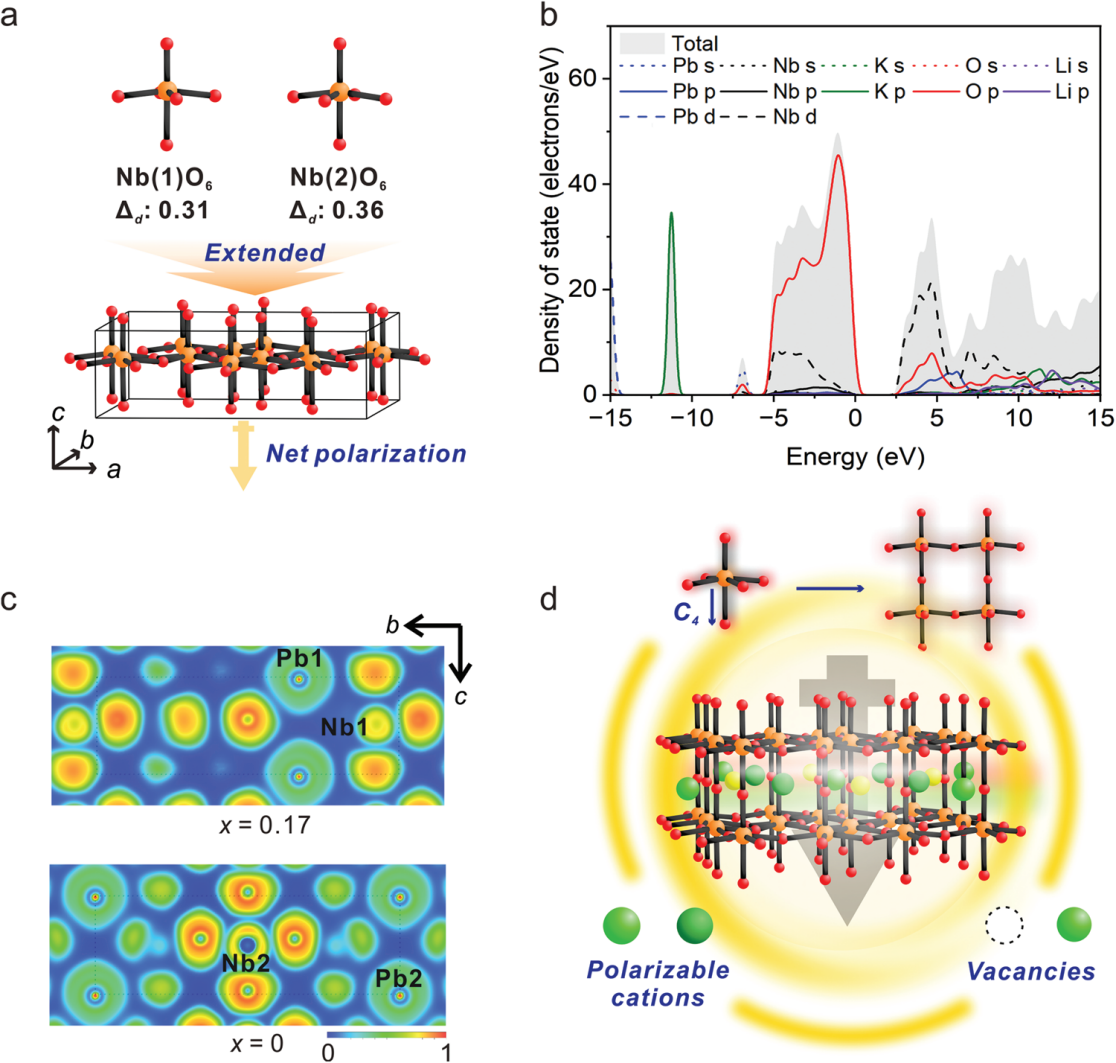
a) Magnitude of out‐of‐center distortion (*Δ*_d_) for distorted Nb(1)O_6_ and Nb(2)O_6_ octahedra and a net moment occurring from the alignment of distorted NbO_6_ octahedra pointing toward the [00−1] direction. b) PDOS for Pb_1.91_K_3.22_□_0.85_Li_2.96_Nb_10_O_30_. c) ELF plots illustrating a sliced‐plane diagrams with *x* = 0.17 and *x* = 0 along the *a*–axis. d) Schematic representation explaining the origin of giant SHG efficiency of Pb_1.91_K_3.22_□_0.85_Li_2.96_Nb_10_O_30_: a synergistically achieved net moment arising from the polar framework with the well‐ordered distortive NbO_6_ octahedra and highly polarizable cations.

To understand the origin of the observed giant SHG efficiency of the title material, several calculation methods have been utilized. First, calculations on the magnitude of out‐of‐center distortion (*Δ*_d_) for Nb(1)O_6_ and Nb(2)O_6_ octahedra result in values of 0.31 and 0.36, respectively (**Figure** **4**a).^[^ ^9^ ^]^ To quantify the intrinsic moments arising from the distorted NbO_6_ octahedra, we further calculated their dipole moments by using a simple bond valence method. By doing so, local dipole moments of 3.42 D and 5.80 D have been obtained for Nb(1)O_6_ and Nb(2)O_6_, respectively. In addition, since all the moments arising from Nb(1)O_6_ and Nb(2)O_6_ octahedra point toward the −*c* direction, a net polarization of 38.08 D occurs along the [00−1] direction from the framework (Figure 4a and **Table** **1**; Figures S6 and S7, Supporting Information). Also, it has been known that TB structures containing vacancies in the channels might enhance local structural distortions.^[^ ^5^, ^10^ ^]^ Thus, the tolerance factor (*t*) calculations have been performed to account for the role of vacancies in the structure.^[^ ^5^, ^10^, ^11^ ^]^ As described before, A site and B site of Pb_1.91_K_3.22_□_0.85_Li_2.96_Nb_10_O_30_ are partially occupied by Pb/□ and Pb/K, respectively. Thus, tolerance factors for the respective sites and the overall material, that is, *t*_Pb_, *t*_Pb/K_, and *t*_total_ are calculated to be 0.779, 0.991, and 0.919, respectively (Table S8, Supporting Information). Here, the structural distortion of the title material with the *t*_total_ value of 0.919 should be attributable to the vacancies in the unfilled sites in the channels, which significantly enhance structural distortions in the title compound.

**Table 1:** Calculated dipole moments of Nb(1)O_6_ and Nb(2)O_6_ octahedra

	Local dipole moments [D]		Net dipole moment [D]	Direction
	Nb(1)O_6_	Nb(2)O_6_		
Pb_1.91_K_3.22_□_0.85_Li_2.96_Nb_10_O_30_	3.42	5.80	38.08	[00−1]

Density functional theory (DFT) calculations were also performed to investigate the relationship between the structure and optical properties. Band structure calculations and partial density of state (PDOS) suggest that Pb_1.91_K_3.22_□_0.85_Li_2.96_Nb_10_O_30_ has a direct band gap of ≈2.64 eV (Figure 4b and Figure S13, Supporting Information), which is smaller than the measured optical band gap because of the discontinuity of exchange–correlation energy.^[^ ^12^ ^]^ While the valence band maximum (VBM) is mainly due to 2p orbitals from O atoms, the conduction band minimum (CBM) includes large contributions from Nb 4d orbitals, O 2p orbitals, and Pb 6p orbitals. The PDOS also indicates that the nonlinear and linear optical properties of Pb_1.91_K_3.22_□_0.85_Li_2.96_Nb_10_O_30_ mostly come from the NbO_6_, PbO_12_, and Pb/KO_15_ polyhedra because the optical properties are closely related to the behavior of the electrons near the VBM and CBM. It is well known that strong interactions between Pb 6s orbitals and O 2p orbitals result in a high contribution of the Pb 6s orbital character in the VBM, forming an asymmetric electron density.^[^ ^13^ ^]^ However, there is little contribution of 6s orbitals from Pb in VBM, suggesting that 6s electrons on Pb are stereochemically inert (Figure 4b). The coordination environment of Pb can be visualized by the electron localization function (ELF) diagrams (Figure 4c and Figure S14, Supporting Information). As can be seen in the ELF diagrams, the limited stereoactivity of lone pairs on Pb^2+^ cations is consistent with the result of PDOS; however, it can be clearly seen that the electron density of highly polarizable Pb^2+^ cations is severely distorted. In addition, strong interatomic interactions between the NbO_6_ octahedra and highly polarizable heavy metal cations such as Pb^2+^ or K^+^ are clearly visualized from the ELF diagrams. Overall, the extremely strong SHG efficiency for the reported material is attributed to the constructive addition of well‐ordered moments arising from the distorted NbO_6_ octahedra, structural distortions in the constituting channels with vacancies, and strong interactions of the framework with highly polarizable cations (Figure 4d).

## 3 Conclusion

A polar TTB, Pb_1.91_K_3.22_□_0.85_Li_2.96_Nb_10_O_30_ has been synthesized by a high temperature solid‐state reaction. XRD analysis indicates that NCS Pb_1.91_K_3.22_□_0.85_Li_2.96_Nb_10_O_30_ exhibits a 3D TTB structure. Pb_1.91_K_3.22_□_0.85_Li_2.96_Nb_10_O_30_ undergoes a reversible phase transition between the NCS (*P*4*bm*) and CS (*P*4/*mbm*) at ≈460 °C, whereas the material goes through an irreversible phase transition to form the thermodynamically stable CS Pb_1.45_K_3.56_Li_3.54_Nb_10_O_30_ (*P*4/*mbm*) upon heating to 1200 °C. Powder SHG measurements using 1064 nm radiation indicate that the new polar NCS Pb_1.91_K_3.22_□_0.85_Li_2.96_Nb_10_O_30_ exhibits a giant type‐I phase‐matchable SHG of 71.5 × KDP that is the largest intensity in the visible range among the reported NLO materials thus far. A closer structural investigation along with several calculation methods suggests that the remarkably strong SHG efficiency for the title material is attributed to the synergistic effect from the highly aligned moments originating from the distorted NbO_6_ octahedra, vacancy‐driven structural distortions in the constituting channels, and strong interactions of the framework with highly polarizable cations. We do believe that the discovery of the novel polar TTB material with the extremely large SHG could further accelerate to open a new way toward various industries utilizing innovative coherent lights.

## 4 Experimental Section

PbO (99%, Sigma Aldrich), K_2_CO_3_ (99%, Daejung), Li_2_CO_3_ (99%, Junsei), Nb_2_O_5_ (99.9%, Junsei), and LiBO_2_ (99.9%, Alfa Aesar) were used as reagents. Polycrystalline sample of Pb_1.91_K_3.22_□_0.85_Li_2.96_Nb_10_O_30_ was synthesized by a conventional solid‐state reaction at high temperature. Stoichiometric amounts of A_2_CO_3_ (A = Li and K; 10% excess), PbO (10% excess), and Nb_2_O_5_ were thoroughly mixed and ground using a mortar and pestle. The homogeneous mixture was loaded into an alumina crucible and heated to 700 °C for 2 h, 900 °C for 4 h, and 1020 °C for 8 h in a muffle furnace and cooled to room temperature. The product was obtained along with very small amount (<1%) of Li_3_NbO_4_ (ICSD‐30246; space group: *I*23) impurity based on the PXRD (Figure S15, Supporting Information).

Although the crystal structure of the related tetragonal TB, Pb_0.91_K_1.72_Li_1.46_Nb_5_O_15_ was previously reported,^[^ ^6^ ^]^ high quality single crystals were grown and the structure of Pb_1.91_K_3.22_□_0.85_Li_2.96_Nb_10_O_30_ was determined to better understand the structure‐NLO properties relationship. Single crystals of the title compound were successfully grown by a high temperature solution method with LiBO_2_ as a flux. The polycrystalline product and LiBO_2_ were initially mixed with 7:3 molar ratio using a mortar and pestle. The homogeneous mixture was transferred to a platinum crucible and heated to 950 °C for 10 h. After heating, the reaction product was slowly cooled to 650 °C at a rate of 3 °C h^−1^, then the furnace was turned off to cool the temperature rapidly to room temperature. After washing extra LiBO_2_ using 2 m HCl solution, colorless rod‐shaped single crystals of Pb_1.91_K_3.22_□_0.85_Li_2.96_Nb_10_O_30_ along with polycrystalline LiNbO_3_ were obtained in ≈97% yield (Figure S16, Supporting Information). Crystals of Pb_1.45_K_3.56_Li_3.54_Nb_10_O_30_ were grown by a high‐temperature solution method. A 0.5 g portion of polycrystalline Pb_1.91_K_3.22_□_0.85_Li_2.96_Nb_10_O_30_ was placed into a platinum crucible and heated to 1200 °C for 60 h and rapidly cooled to room temperature by turning the furnace off. After cooling, colorless rod‐shaped single crystals were obtained in ≈98% yield along with polycrystalline LiNbO_3_ (Figure S17, Supporting Information).

Crystal structures for the reported materials have been determined by SC‐XRD. Colorless rod single crystals with the size of 0.077 mm × 0.125 mm × 0.255 mm for Pb_1.91_K_3.22_□_0.85_Li_2.96_Nb_10_O_30_ (*P*4*bm*) and 0.055 mm × 0.103 mm × 0.135 mm for Pb_1.45_K_3.56_Li_3.54_Nb_10_O_30_ (*P*4/*mbm*) were selected for the SC‐XRD measurements. The SC‐XRD data were collected by using a Bruker D8 QUEST diffractometer with graphite monochromated Mo K*α* radiation (*λ* = 0.71703 Å) at the Advanced Bio‐Interface Core Research Facility, Sogang University. The data reduction and absorption correction for the obtained data were performed through the SAINT^[^ ^14^ ^]^ and SADABS^[^ ^15^ ^]^ software, respectively. The crystal structures were solved using SHELXS‐2013^[^ ^16^ ^]^ and refined by SHELXL‐2013^[^ ^17^ ^]^ implemented in WinGX‐2014.^[^ ^18^ ^]^ PXRD data were obtained by a Rigaku MiniFlex 600 with 40 kV and 15 mA using Cu K*α* (*λ* = 1.5406Å) radiation at room temperature. Temperature‐dependent in situ PXRD patterns were collected using the benchtop heating stage (BTS‐500, Anton‐Paar) at the temperature range of 25–500 °C. Synchrotron powder diffraction pattern was collected on the 2D supramolecular crystallography beamline in Pohang Acceleration Laboratory at room temperature in the 2*θ* range of 3°–65° using synchrotron radiation (*λ* = 0.68880 Å). The Rietveld refinements were performed by using GSAS software^[^ ^19^ ^]^ with an initial model obtained from the SC‐XRD data. Final Rietveld refinement plots and the detail crystallographic results for the title compounds are in Figures S18,  S19 and Tables S1–S3, Supporting Information, respectively.

IR spectrum was obtained by a Thermo Fisher Scientific Nicolet iS50 spectrometer in the range of 400–4000 cm^−1^ at room temperature. The ground sample was placed on the diamond attenuated total reflectance crystal.

UV–vis spectrum was recorded on a JASCO V‐650 in the range of 200–700 nm at room temperature. The band gap for the title compound was calculated using the Kubelka‐Munk equation.^[^ ^8^ ^]^

TGA and DSC measurements were performed using a SCINCO TGA N‐1000 and TA Q2000 DSC thermal analyzer, respectively. The polycrystalline sample was placed on an alumina crucible and heated to 900 °C (500 °C for DSC) at a rate of 10 °C min^−1^ under flowing Ar.

Field emission scanning electron microscopy and EDX Spectroscopy (FE‐SEM/EDX) results were collected by using a JEOL Benelux JSM‐7100F attached to an Oxford Instruments NanoAnalysis AZtecEnergy. The observed heavier atoms’ ratio from the EDX results for Pb_1.91_K_3.22_□_0.85_Li_2.96_Nb_10_O_30_ matches well with the XRD experiments (Table S9, Supporting Information).

The quantitative analysis for Li^+^ and K^+^ was conducted through an ICP‐OES using an Agilent ICP‐OES 5900 spectrometer. The title compound was dissolved in a hydrofluoric acid and nitric acid for ICP‐OES measurements. Calculated (experimental): Li^+^, 1.05% (1.14%); K^+^, 6.45% (6.74%) (Table S9, Supporting Information).

^7^Li solid‐state MAS NMR analysis was performed on a Bruker AVANCE III HD 400 MHz in a 9.4 T magnetic field at the Western Seoul Center, Korea Basic Science Institute. The polycrystalline sample was placed in a 4 mm HXY‐MAS probe with a sample spinning frequency of 10 kHz. The ^7^Li MAS NMR spectrum was collected at a Larmor frequency of 155.506 MHz with a delay of 10 s. Chemical shift was referenced to aqueous LiCl solution at 0 ppm.

DFT calculations were performed to investigate the relationship between the structure and optical properties of the title compound. Because the crystal structure contains disordered Pb/K sites, a supercell software was employed to generate an appropriate supercell structure.^[^ ^20^ ^]^ The band structure and DOS were calculated using the CASTEP package,^[^ ^21^ ^]^ which employs norm‐conserving pseudopotentials and the Perdew–Burke–Ernzerhof functionals for all elements (Pb, K, Li, Nb, and O).^[^ ^22^ ^]^ An energy cutoff of ≈925.20 eV was used, and the total energy convergence threshold was set to 10^−6^ Ry. The Brillouin zone was sampled with a *k*‐point separation of 0.03 Å^−1^. ELF plots were calculated using the Quantum Espresso package^[^ ^23^ ^]^ and visualized with the VESTA program.^[^ ^24^ ^]^

Powder SHG measurements were performed through a modified Kurtz–Perry nonlinear optical system.^[^ ^25^ ^]^ After sieving into distinct particle sizes, each graded sample was packed into capillary tubes (o.d. = 2.0 mm and i.d. = 1.8 mm) and irradiated by using DAWA Q‐switched Nd:YAG laser (1064 nm). The SHG light (532 nm) was detected by a Hamamatsu photomultiplier tube and monitored by a Tektronix TDS 1012 oscilloscope.

The stability of the TB structure has been estimated by the tolerance factor (*t*) as in the case of perovskite.^[^ ^11^, ^26^ ^]^ Pb_1.91_K_3.22_□_0.85_Li_2.96_Nb_10_O_30_ has two sites for heavy metal cations, namely, the quadrangular Pb sites and pentagonal Pb/K sites. The individual tolerance factor for Pb and Pb/K sites can be calculated by using the following equations:

(1) $$t_{\mathrm{Pb}}\;=\;\frac{\left(r_{\mathrm{Pb}}\;+\;r_{O}\right)}{\sqrt{2}\left(r_{\mathrm{Nb}}\;+\;r_{O}\right)}$$

(2) $$t_{\mathrm{Pb}/K}=\;\frac{\left(r_{\mathrm{Pb}/K}+r_{O}\right)}{\sqrt{23-12\sqrt{3}}\left(r_{\mathrm{Nb}}+r_{O}\right)}$$

(3) $$t_{\mathrm{total}}=\;\frac{\left(t_{\mathrm{Pb}}+2t_{\mathrm{Pb}/K}\right)}{3}$$

where *t*_Pb_, *t*_Pb/K_, and *t*_total_ are the geometric tolerance factors, *r*_Pb_, *r*_Pb/K_, *r*_Nb_, and *r*_O_ are the Shannon's ionic radii^[^ ^27^ ^]^ for Pb, Pb/K, Nb, and O, respectively. A *t*_total_ value of 1 assumes no structural distortions in the framework.

Further details of the crystal structure investigations may be obtained from the Fachinformationszentrum Karlsruhe, 76344 Eggenstein‐Leopoldshafen (Germany), on quoting the depository numbers CSD‐2239720‐2239721.

## Conflict of Interest

The authors declare no conflict of interest.

## Supporting information

Supporting Information

Supporting Information

## Data Availability

The data that support the findings of this study are available from the corresponding author upon reasonable request.
