# Supplementary material for: A Polar Tetragonal Tungsten Bronze with Colossal Second‐Harmonic Generation
Source: Adv Sci (Weinh). 2023 Apr 23;10(19):2301374. doi: 10.1002/advs.202301374 (PMC10323606; doi:10.1002/advs.202301374)
Supplement: Supplementary file 1 — Supporting Information [file ADVS-10-2301374-s001.pdf]

ADVANCED SCIENCE
Open Access

Supporting Information

for *Adv. Sci.*, DOI 10.1002/advs.202301374

A Polar Tetragonal Tungsten Bronze with Colossal Second-Harmonic Generation

*Yunseung Kuk, Seong Bin Bae, Sang Mo Yang and Kang Min Ok**

Supporting Information

A Polar Tetragonal Tungsten Bronze with Colossal Second-Harmonic Generation

*Yunseung Kuk, Seong Bin Bae, Sang Mo Yang, and Kang Min Ok**

Contents

Sections	Titles	Pages
**Table S1.**	Crystallographic table for $Pb_{1.91}K_{3.22}\square_{0.85}Li_{2.96}Nb_{10}O_{30}$ and $Pb_{1.45}K_{3.56}Li_{3.54}Nb_{10}O_{30}$.	S4
**Table S2.**	Selected distances (Å) for $Pb_{1.91}K_{3.22}\square_{0.85}Li_{2.96}Nb_{10}O_{30}$.	S5
**Table S3.**	Atomic coordinates, displacement parameters, and occupancy data for $Pb_{1.91}K_{3.22}\square_{0.85}Li_{2.96}Nb_{10}O_{30}$.	S6
**Table S4.**	Calculated dipole moments of Nb(1)O_6 and Nb(2)O_6 octahedra for $Pb_{1.45}K_{3.56}Li_{3.54}Nb_{10}O_{30}$.	S7
**Table S5.**	Comparison of the SHG efficiencies and band gap among the reported NLO materials.	S8
**Table S6.**	Calculated dipole moments of Nb(1)O_6, Nb(2)O_6, PbO_{12}, and Pb/KO_{15} polyhedra for $Pb_{1.91}K_{3.22}\square_{0.85}Li_{2.96}Nb_{10}O_{30}$.	S9
**Table S7.**	Comparison of the magnitude and direction of dipole moments among the reported tungsten bronze structures.	S10
**Table S8.**	Comparison of the geometric tolerance factor and SHG efficiencies among the reported tungsten bronze structures.	S11
**Table S9.**	Weight and atomic ratio comparison for $Pb_{1.91}K_{3.22}\square_{0.85}Li_{2.96}Nb_{10}O_{30}$ and $Pb_{0.91}K_{1.72}Li_{1.46}Nb_5O_{15}$: ICP-OES and EDX results.	S12
**Figure S1.**	Asymmetric unit of $Pb_{1.91}K_{3.22}\square_{0.85}Li_{2.96}Nb_{10}O_{30}$.	S13
**Figure S2.**	FE-SEM/EDX result for $Pb_{1.91}K_{3.22}\square_{0.85}Li_{2.96}Nb_{10}O_{30}$.	S14
**Figure S3.**	^{7}Li solid-state MAS NMR spectrum for $Pb_{1.91}K_{3.22}\square_{0.85}Li_{2.96}Nb_{10}O_{30}$.	S15
**Figure S4.**	IR spectrum for $Pb_{1.91}K_{3.22}\square_{0.85}Li_{2.96}Nb_{10}O_{30}$.	S16
**Figure S5.**	UV-vis spectrum for $Pb_{1.91}K_{3.22}\square_{0.85}Li_{2.96}Nb_{10}O_{30}$. (a) Kubelka-Munk and (b) Tauc plot with direct allowed band transition (n=2).	S17
**Figure S6.**	TGA diagram for $Pb_{1.91}K_{3.22}\square_{0.85}Li_{2.96}Nb_{10}O_{30}$.	S18
**Figure S7.**	Temperature-dependent PXRD patterns of $Pb_{1.91}K_{3.22}\square_{0.85}Li_{2.96}Nb_{10}O_{30}$ in the 2θ range of (a) 5–70°, (b) 20–25°, (c) 27.5–32.5°, and (d) 44–46°, respectively.	S19

Figure S8. Final Rietveld refinement plot for $Pb_{1.91}K_{3.22}\square_{0.85}Li_{2.96}Nb_{10}O_{30}$ at 480 °C. S20

Figure S9. PXRD pattern of $Pb_{1.91}K_{3.22}\square_{0.85}Li_{2.96}Nb_{10}O_{30}$ at 480 °C. (inset) Magnified PXRD pattern in the range of 21.5–32.5°. The black and red bars represent the Bragg positions of *P*4/*mbm* and *P*4*bm* phase, respectively. S21

Figure S10. PXRD patterns of $Pb_{1.91}K_{3.22}\square_{0.85}Li_{2.96}Nb_{10}O_{30}$ (NCS phase; S.G.: *P*4*bm*) and $Pb_{1.45}K_{3.56}Li_{3.54}Nb_{10}O_{30}$ (CS phase; S.G.: *P*4/*mbm*). S22

Figure S11. Ball-and-stick and polyhedral representation of Nb(1)O_6 and Nb(2)O_6 octahedra for $Pb_{1.45}K_{3.56}Li_{3.54}Nb_{10}O_{30}$. S23

Figure S12. (a) Calculated and experimental PXRD patterns for Li_3NbO_4. (b) Oscilloscope traces of the SHG signals of α-SiO_2, Li_3NbO_4, and KDP in the particle size range of 20–45 µm. S24

Figure S13. Band structure for $Pb_{1.91}K_{3.22}\square_{0.85}Li_{2.96}Nb_{10}O_{30}$. Black arrow indicates the optical transition from the valence band maximum (VBM) to the conduction band minimum (CBM). S25

Figure S14. Ball-and-stick models and electron localization function (ELF) diagrams for $Pb_{1.91}K_{3.22}\square_{0.85}Li_{2.96}Nb_{10}O_{30}$ along the *c*-axis with (a) $z = 0$ and (b) $z = 0.5$ (dark green, Pb/K; light green, Pb; yellow, Li; orange, Nb; red, O). S26

Figure S15. Calculated and experimental PXRD patterns for $Pb_{1.91}K_{3.22}\square_{0.85}Li_{2.96}Nb_{10}O_{30}$. S27

Figure S16. PXRD patterns for $Pb_{1.91}K_{3.22}\square_{0.85}Li_{2.96}Nb_{10}O_{30}$ and the melted sample. S28

Figure S17. Calculated and experimental PXRD patterns for $Pb_{1.45}K_{3.56}Li_{3.54}Nb_{10}O_{30}$. S29

Figure S18. Final Rietveld refinement plot for $Pb_{1.91}K_{3.22}\square_{0.85}Li_{2.96}Nb_{10}O_{30}$. S30

Figure S19. Final Rietveld refinement plot of synchrotron powder X-ray diffraction pattern (λ = 0.68880 Å) for $Pb_{1.91}K_{3.22}\square_{0.85}Li_{2.96}Nb_{10}O_{30}$. S31

Figure S20. Oscilloscope traces of the SHG signals of $Pb_{1.91}K_{3.22}\square_{0.85}Li_{2.96}Nb_{10}O_{30}$ measured under different laser power. S32

Reference S33

Table S1. Crystallographic table for $Pb_{1.91}K_{3.22}\square_{0.85}Li_{2.96}Nb_{10}O_{30}$ and $Pb_{1.45}K_{3.56}Li_{3.54}Nb_{10}O_{30}$.

Parameter				
Formula	$Pb_{1.91}K_{3.22}\square_{0.85}Li_{2.96}Nb_{10}O_{30}$	$Pb_{1.91}K_{3.22}\square_{0.85}Li_{2.96}Nb_{10}O_{30}$	$Pb_{1.91}K_{3.22}\square_{0.85}Li_{2.96}Nb_{10}O_{30}$	$Pb_{1.45}K_{3.56}Li_{3.54}Nb_{10}O_{30}$
F.W.	1951.28	1951.28	1951.28	1872.93
Space group	*P*4*bm*	*P*4*bm*	*P*4*bm*	*P*4/*mbm*
a (Å)	12.533(5)	12.53949(5)	12.53291(12)	12.5893(5)
b (Å)	12.533(5)	12.53949(5)	12.53291(12)	12.5893(5)
c (Å)	4.016(2)	4.03846(2)	4.03436(5)	3.9612(2)
V (Å^3)	630.9(6)	635.003(7)	633.692(17)	627.81(6)
Z	1	1	1	1
λ (Å)	0.71073	1.5406	0.68880	0.71703
T (K)	297(2)	297(2)	297(2)	297(2)
$R(F_o)^a$ or R_p^b	0.0268	0.0545	0.0132	0.0329
$R_w(F_o^2)^c$ or R_{wp}^d	0.0619	0.0769	0.0177	0.0653
Flack *x*	0.49(3)	-	-	-

$^aR(F) = \sum||F_o| - |F_c|| / \sum|F_o|$. $^bR_p = \sum|I_o - I_c| / \sum I_o$.

$^cR_w(F_o^2) = [\sum w(F_o^2 - F_c^2)^2 / \sum w(F_o^2)^2]^{1/2}$. $^dR_{wp} = [\sum w(I_o - I_c)^2 / \sum w I_o^2]^{1/2}$.

Table S2. Selected distances (Å) for $Pb_{1.91}K_{3.22}\square_{0.85}Li_{2.96}Nb_{10}O_{30}$.

Nb(1)–O(1)	2.17(3)	Pb(1)/K(1)–O(5)	2.714(10)
Nb(1)–O(1)	1.86(3)	Pb(1)/K(1)–O(5)	3.162(11)
Nb(1)–O(2)	2.0060(17)	Pb(1)/K(1)–O(5)	2.714(10)
Nb(1)–O(4)	1.961(4)	Pb(2)–O(1)	2.803(5)
Nb(1)–O(4)	2.004(4)	Pb(2)–O(1)	2.803(5)
Nb(1)–O(5)	1.959(3)	Pb(2)–O(1)	2.803(5)
Nb(2)–O(3)	2.20(5)	Pb(2)–O(1)	2.803(5)
Nb(2)–O(3)	1.84(5)	Pb(2)–O(4)	3.019(10)
Nb(2)–O(5)	1.975(4)	Pb(2)–O(4)	2.613(9)
Nb(2)–O(5)	1.975(4)	Pb(2)–O(4)	3.019(10)
Nb(2)–O(5)	1.975(4)	Pb(2)–O(4)	2.613(9)
Nb(2)–O(5)	1.975(4)	Pb(2)–O(4)	3.019(10)
Pb(1)/K(1)–O(1)	3.451(5)	Pb(2)–O(4)	2.613(9)
Pb(1)/K(1)–O(1)	3.232(5)	Pb(2)–O(4)	3.019(10)
Pb(1)/K(1)–O(1)	3.232(5)	Pb(2)–O(4)	2.613(9)
Pb(1)/K(1)–O(1)	3.451(5)	Li(1)–O(1)	2.193(13)
Pb(1)/K(1)–O(2)	3.114(10)	Li(1)–O(1)	2.193(13)
Pb(1)/K(1)–O(2)	2.709(8)	Li(1)–O(2)	2.61(17)
Pb(1)/K(1)–O(3)	3.001(3)	Li(1)–O(2)	2.57(17)
Pb(1)/K(1)–O(4)	3.576(12)	Li(1)–O(3)	2.16(2)
Pb(1)/K(1)–O(4)	3.198(10)	Li(1)–O(5)	2.62(17)
Pb(1)/K(1)–O(4)	3.576(12)	Li(1)–O(5)	2.52(17)
Pb(1)/K(1)–O(4)	3.198(10)	Li(1)–O(5)	2.62(17)
Pb(1)/K(1)–O(5)	3.162(11)	Li(1)–O(5)	2.52(17)

Table S3. Atomic coordinates, displacement parameters, and occupancy data for $Pb_{1.91}K_{3.22}\square_{0.85}Li_{2.96}Nb_{10}O_{30}$.

Atoms	Wyck.	*x*	*y*	*z*	U_{iso}	Occupancy
Nb(1)	8d	0.07434(5)	0.20922(5)	0.632(2)	0.00711(15)	1.0
Nb(2)	2b	0	0.5	0.659(4)	0.0112(4)	1.0
K(1)	4c	-0.16903(9)	0.33097(9)	0.15237(9)	0.0564(9)	0.8057(13)
Pb(1)	4c	-0.16903(9)	0.33097(9)	0.15237(9)	0.0564(9)	0.1943 (13)
Pb(2)	2a	0	0	0.144(4)	0.0112(5)	0.5711(12)
O(1)	8d	0.0771(3)	0.2092(4)	1.094(9)	0.0278(9)	1.0
O(2)	4c	0.2133(2)	0.2867(2)	0.580(3)	0.0098(9)	1.0
O(3)	2b	0	0.5	1.115(14)	0.0408(9)	1.0
O(4)	8d	0.1399(2)	0.0685(2)	0.574(5)	0.0105(9)	1.0
O(5)	8d	-0.0005(2)	0.3450(2)	0.572(4)	0.0152(9)	1.0
Li(1)	4c	-0.1217(14)	0.6217(14)	1.09(6)	0.045(15)	0.74(4)

Table S4. Calculated dipole moments of $Nb(1)O_6$ and $Nb(2)O_6$ octahedra for $Pb_{1.45}K_{3.56}Li_{3.54}Nb_{10}O_{30}$.

Polyhedra	Direction of dipole moments (Debyes)			Magnitude
	x	*y*	*z*	
$Nb(1)O_6$	-0.2303	0.8808	0	0.91
	-0.8807	-0.2303	0	0.91
	-0.2303	-0.8808	0	0.91
	0.2303	0.8808	0	0.91
	0.2303	-0.8808	0	0.91
	0.8807	-0.2303	0	0.91
	-0.8808	0.2303	0	0.91
	0.8808	0.2303	0	0.91
$Nb(2)O_6$	0	0	0	0
	0	0	0	0
Total	0	0	0	0

Table S5. Comparison of the SHG efficiencies and band gap among the reported NLO materials.

Entry	Space group	SHG efficiencies	Band gap	Structure type
$Pb_{1.91}K_{3.22}\square_{0.85}Li_{2.96}Nb_{10}O_{30}$[a]	*P4bm*	71.5 × KDP[b]	3.45 eV	Tungsten bronze
$Pb_{2.15}(Li_{0.25}Na_{0.75})_{0.7}Nb_5O_{15}$[[1]]	*Bb*2$_1$*m*	47 × KDP	2.83 eV	Tungsten bronze
$Pb_{2.15}Li_{0.6}Nb_5O_{15}$[[2]]	*Pn*2$_1$*m*	44 × KDP	X[c]	Tungsten bronze
$Pb_2(Pb_{0.15}Li_{0.7}\square_{0.15})Nb_5O_{15}$[[2]]	*Pn*2$_1$*m*	39 × KDP	2.84 eV	Tungsten bronze
$Pb_2RNb_5O_{15}$ (R: Na, K, and Rb)[[2]]	*Cm*2*m*	18–28 × KDP	X	Tungsten bronze
$Pb_2AgNb_5O_{15}$[[3]]	*Cm*2*m*	0.2 × $LiNbO_3$	2.64 eV	Tungsten bronze
$PbBiNb_5O_{15}$[[4]]	*Cm*2*m*	0.3 × KDP	X	Tungsten bronze
$A_6M_2Nb_8O_{30}$ (A: Sr, Ba, and Pb; M: Ti, Zr, or Hf)[[5]]	*Pba*2, *P*4*bm*, *Cm*2*m*	20–600 × α-SiO_2	X	Tungsten bronze
$A(MoO_2)_2O(IO_4)$ (A: Rb and Cs)[[6]]	*P*2	30–32 × KDP	3.31–3.33 eV	HTO[d]
$AGa_3F_6(SeO_3)_2$ (A: Rb and Cs)[[7]]	*P*6$_3$*mc*	5.4–5.6 × KDP	3.57–3.65 eV	HTO
$A_2(MoO_3)_3(SeO_3)$ (A: Rb and Tl)[[8]]	*P*6$_3$, *P*31*c*	300–400 × α-SiO_2	3.00–3.20 eV	HTO
Pb_2BO_3I[[9]]	*P*321	10 × KDP	2.90 eV	KBBF-family[e]
Pb_2BO_3Br[[10]]	*P*321	9.5 × KDP	3.33 eV	KBBF-family
Pb_2BO_3Cl[[11]]	*P*321	9 × KDP	3.99 eV	KBBF-family

[a] □: Vacancies, [b] KDP: Potassium dihydrogen phostphate, [c]X: Not measured, [d]HTO: Hexagonal tungsten oxide [e]KBBF: KBe_2BO_3F.

Table S6. Calculated dipole moments of $Nb(1)O_6$, $Nb(2)O_6$, PbO_{12}, and Pb/KO_{15} polyhedra for $Pb_{1.91}K_{3.22}\square_{0.85}Li_{2.96}Nb_{10}O_{30}$.

Polyhedra	Direction of dipole moments (Debyes)			Magnitude	Direction
	x	*y*	*z*		
$Nb(1)O_6$	0.108	-0.833	-3.31	3.42	[00−1]
	-0.108	0.833	-3.31	3.42	
	0.833	-0.108	-3.31	3.42	
	-0.833	0.108	-3.31	3.42	
	0.108	0.833	-3.31	3.42	
	-0.108	-0.833	-3.31	3.42	
	0.833	0.108	-3.31	3.42	
	-0.833	-0.108	-3.31	3.42	
Sum	0	0	-26.48	26.48	
$Nb(2)O_6$	0	0	-5.80	5.80	[00−1]
	0	0	-5.80	5.80	
Sum	0	0	-11.60	11.60	
PbO_{15}	0.0797	0.0797	0.114	0.160	[001]
	-0.0797	0.0797	0.114	0.160	
	0.0797	-0.0797	0.114	0.160	
	-0.0797	-0.0797	0.114	0.160	
Sum (Pb(1) Occ: 0.1943)[a]	0	0	0.460	0.460	
KO_{15}	-0.434	-0.434	-4.40	4.45	[00−1]
	0.434	-0.434	-4.40	4.45	
	-0.434	0.434	-4.40	4.45	
	0.434	0.434	-4.40	4.45	
Sum (K(1) Occ: 0.8057)	0	0	-17.60	17.60	
PbO_{12}	0	0	0.655	0.655	[001]
	0	0	0.655	0.655	
Sum (Pb(2) Occ: 0.5711)	0	0	1.310	1.310	

[a]Occ: Occupancy.

Table S7. Comparison of the magnitude and direction of dipole moments among the reported tungsten bronze structures.

Entry	Dipole moments for each polyhedra (D)	Magnitude	Cell volume (A^3)	Moments per volume (esu cm $Å^{-3}$)	Direction
	NbO_6				
$Pb_{1.91}K_{3.22}□_{0.85}Li_{2.96}Nb_{10}O_{30}$	-38.08	38.08	630.9	6.04×10^{-20}	[00−1]
$Pb_2(Pb_{0.15}Li_{0.7}□_{0.15})Nb_5O_{15}$[[2]]	-61.09	61.09	1156.2	5.08×10^{-20}	[0−10]
$Pb_2KNb_5O_{15}$[[2] and [12]]	-71.64	71.64	1251.7	5.72×10^{-20}	[0−10]

Table S8. Comparison of the geometric tolerance factor and SHG efficiencies among the reported tungsten bronze structures.

Entry	Space group	A sites	B sites	t_A	t_B	t_{total}	SHG (× KDP)
$Pb_{1.91}K_{3.22}\square_{0.85}Li_{2.96}Nb_{10}O_{30}$	*P4bm*	Pb: 0.57<br>□: 0.43	Pb: 0.20<br>K: 0.80	0.779	0.991	0.919	71.5
$Pb_2(Pb_{0.15}Li_{0.7}\square_{0.15})Nb_5O_{15}$[[2]]	$Pn2_1m$	Pb: 0.15<br>□: 0.15<br>Li: 0.70	Pb: 1	0.786	0.952	0.897	39
$Pb_2KNb_5O_{15}$[[2] and [12]]	*Cm2m*	Pb: 1	Pb: 0.5<br>K: 0.5	1	0.976	0.985	25

Table S9. Weight and atomic ratio comparison for $Pb_{1.91}K_{3.22}\square_{0.85}Li_{2.96}Nb_{10}O_{30}$ and $Pb_{0.91}K_{1.72}Li_{1.46}Nb_5O_{15}$: ICP-OES and EDX results.

Entry	ICP-OES (wt%)		EDX (at%)		
	K	Li	Pb	K	Nb
Experimental data	6.74	1.14	3.70	6.67	18.76
$Pb_{1.91}K_{3.22}\square_{0.85}Li_{2.96}Nb_{10}O_{30}$	6.45	1.05	1.91	3.22	10
$Pb_{0.91}K_{1.72}Li_{1.46}Nb_5O_{15}$	6.95	1.04	0.91	1.72	5

The EDX analysis of a single crystal revealed the presence of Pb, K, and Nb with normalized heavier atomic ratios of 1 : 1.80 : 5.07, which align more closely with the stoichiometric ratio of the refined formula $Pb_{1.91}K_{3.22}\square_{0.85}Li_{2.96}Nb_{10}O_{30}$ (1 : 1.69 : 5.24) than with $Pb_{0.91}K_{1.72}Li_{1.46}Nb_5O_{15}$ (1 : 1.89 : 5.49).

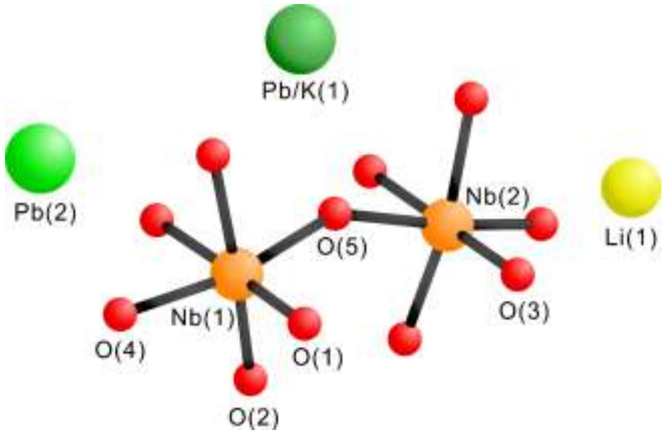

Figure S1. Asymmetric unit of $Pb_{1.91}K_{3.22}\square_{0.85}Li_{2.96}Nb_{10}O_{30}$.

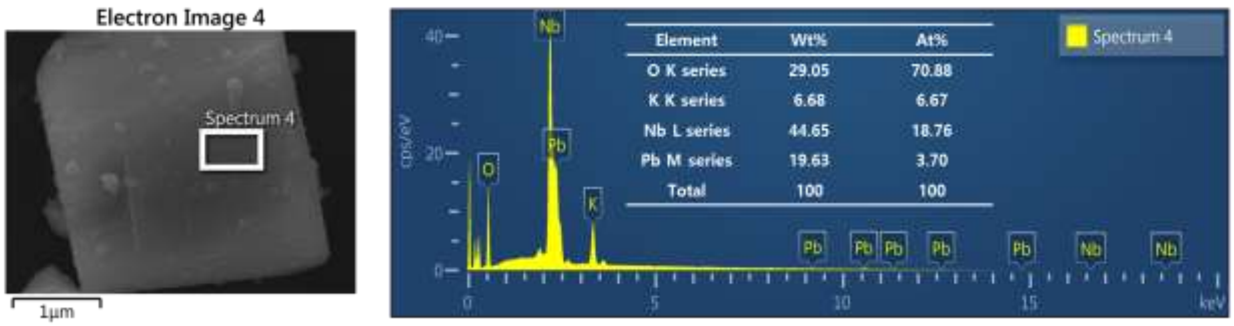

Figure S2. FE-SEM/EDX result for $Pb_{1.91}K_{3.22}\square_{0.85}Li_{2.96}Nb_{10}O_{30}$.

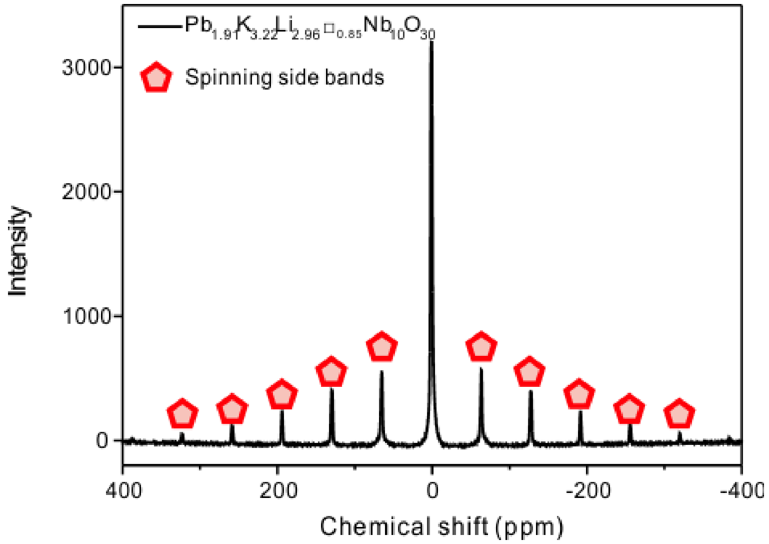

Figure S3. ^{7}Li solid-state MAS NMR spectrum for $Pb_{1.91}K_{3.22}\square_{0.85}Li_{2.96}Nb_{10}O_{30}$.

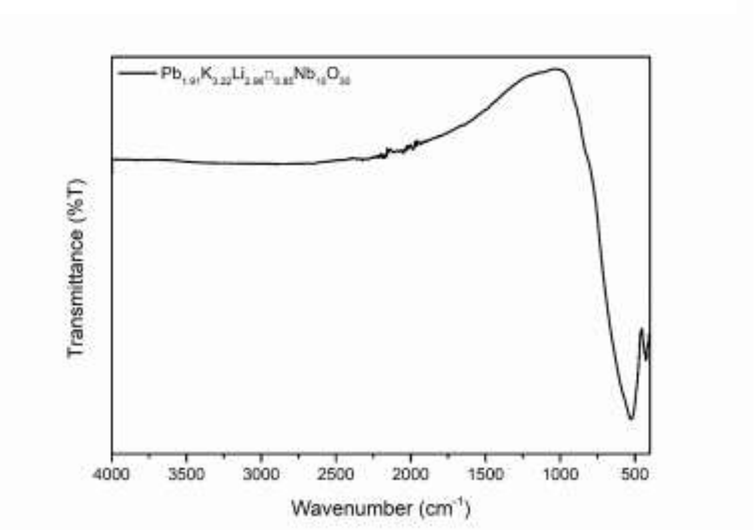

Figure S4. IR spectrum for $Pb_{1.91}K_{3.22}\square_{0.85}Li_{2.96}Nb_{10}O_{30}$.

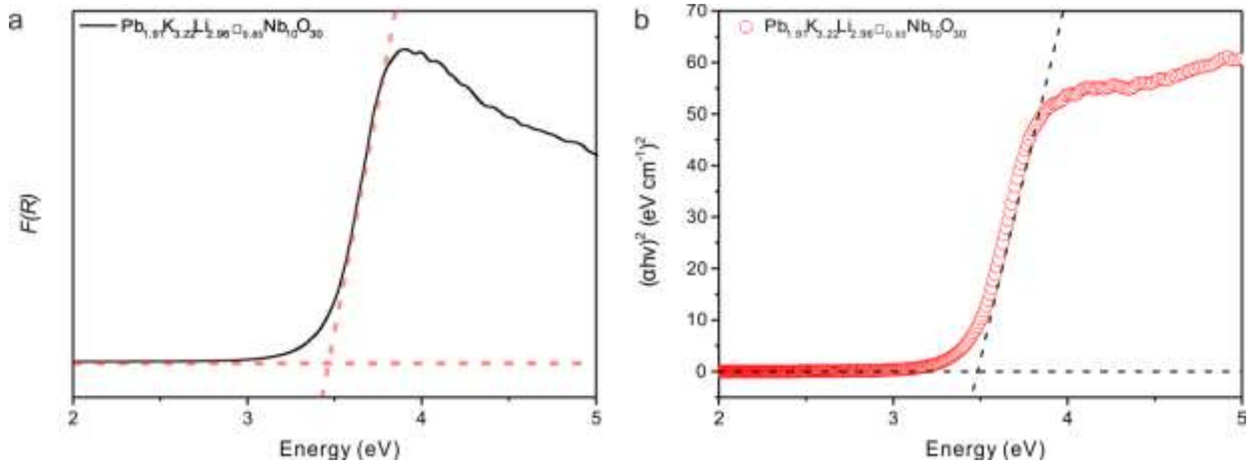

Figure S5. UV-vis spectrum for $Pb_{1.91}K_{3.22}\square_{0.85}Li_{2.96}Nb_{10}O_{30}$. (a) Kubelka-Munk and (b) Tauc plot with direct allowed band transition (n=2).

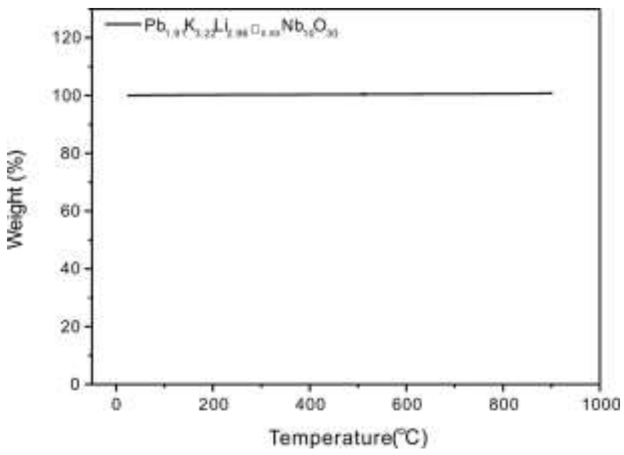

Figure S6. TGA diagram for $Pb_{1.91}K_{3.22}\square_{0.85}Li_{2.96}Nb_{10}O_{30}$.

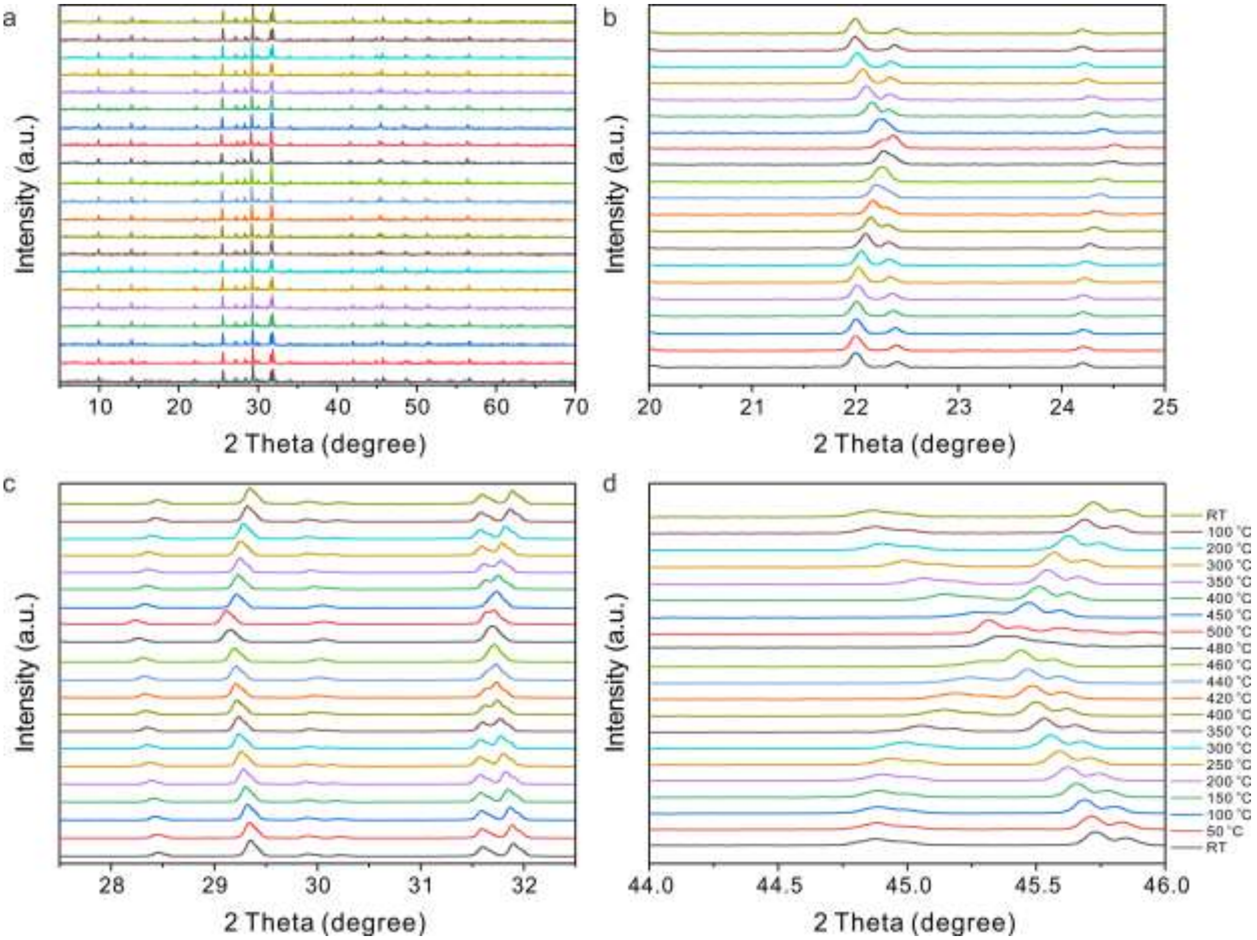

Figure S7. Temperature-dependent PXRD patterns of $Pb_{1.91}K_{3.22}\square_{0.85}Li_{2.96}Nb_{10}O_{30}$ in the 2θ range of (a) 5–70°, (b) 20–25°, (c) 27.5–32.5°, and (d) 44–46°, respectively.

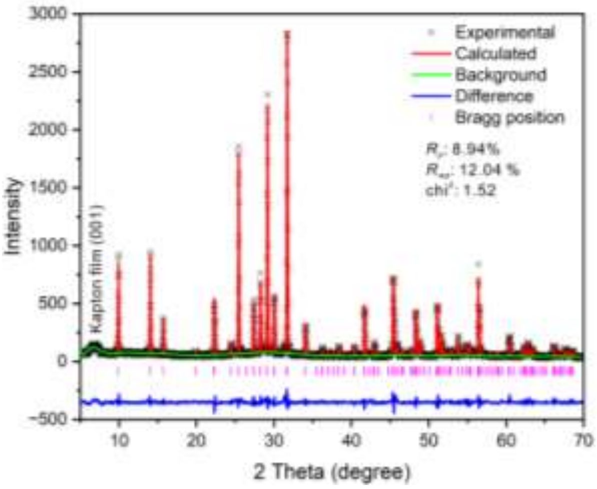

Figure S8. Final Rietveld refinement plot for $Pb_{1.91}K_{3.22}\square_{0.85}Li_{2.96}Nb_{10}O_{30}$ at 480 °C.

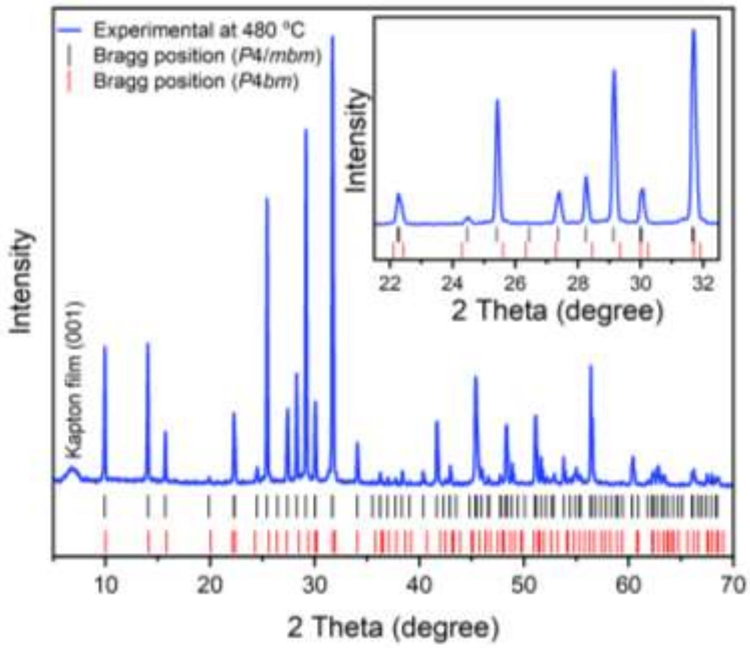

Figure S9. PXRD pattern of $Pb_{1.91}K_{3.22}\square_{0.85}Li_{2.96}Nb_{10}O_{30}$ at 480 °C. (inset) Magnified PXRD pattern in the range of 21.5–32.5°. The black and red bars represent the Bragg positions of *P*4/*mbm* and *P*4*bm* phase, respectively.

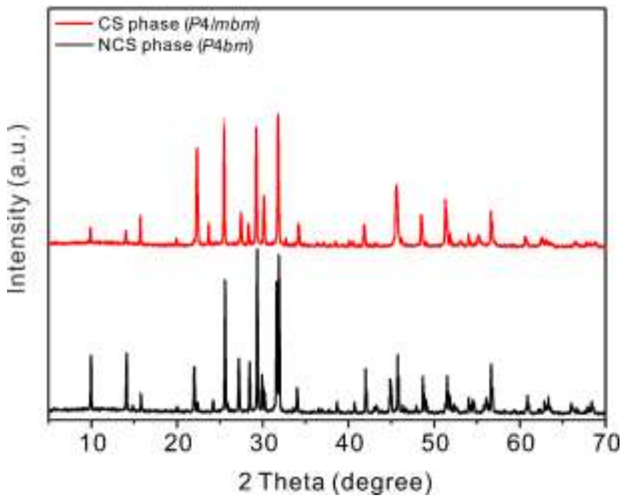

Figure S10. PXRD patterns of $Pb_{1.91}K_{3.22}\square_{0.85}Li_{2.96}Nb_{10}O_{30}$ (NCS phase; S.G.: *P*4*bm*) and $Pb_{1.45}K_{3.56}Li_{3.54}Nb_{10}O_{30}$ (CS phase; S.G.: *P*4/*mbm*).

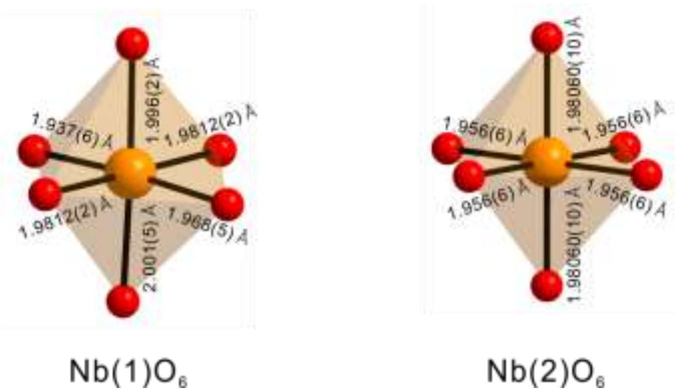

Figure S11. Ball-and-stick and polyhedral representation of $Nb(1)O_6$ and $Nb(2)O_6$ octahedra for $Pb_{1.45}K_{3.56}Li_{3.54}Nb_{10}O_{30}$.

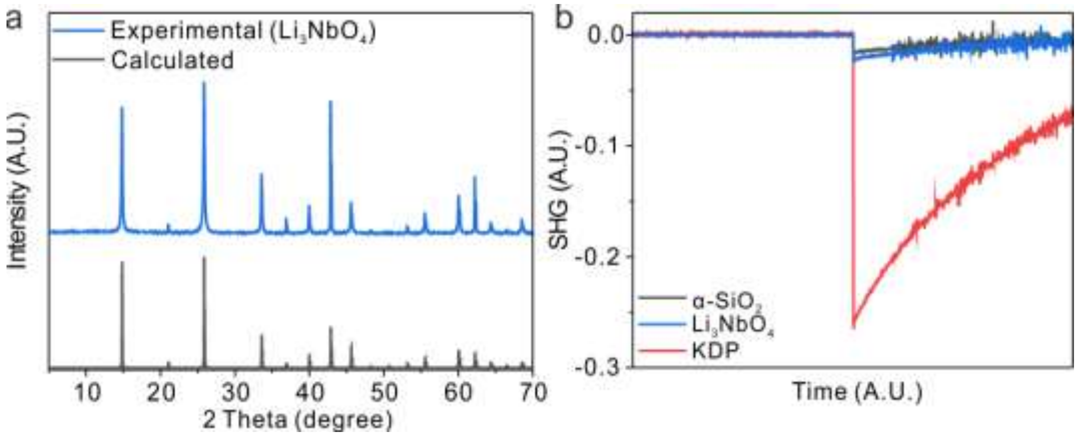

Figure S12. (a) Calculated and experimental PXRD patterns for Li_3NbO_4. (b) Oscilloscope traces of the SHG signals of α-SiO_2, Li_3NbO_4, and KDP in the particle size range of 20–45 μm.

The title compound, $Pb_{1.91}K_{3.22}\square_{0.85}Li_{2.96}Nb_{10}O_{30}$, was obtained along with a very small amount (<1%) of Li_3NbO_4 impurity, as determined by PXRD analysis. Since Li_3NbO_4 also crystallizes in the NCS space group, *I*23, SHG measurements were conducted to investigate if the presence of Li_3NbO_4 has any impact on the SHG of NCS $Pb_{1.91}K_{3.22}\square_{0.85}Li_{2.96}Nb_{10}O_{30}$. Polycrystalline samples of Li_3NbO_4 were synthesized using solid-state reaction,[13] and the experimental pattern (Figure S12a) matched well with the simulated pattern based on the SCXRD result (ICSD-30246). The sieved samples in the particle size range of 200–250 μm were used to measure the SHG intensity. The SHG results indicate that Li_3NbO_4 exhibits a very weak SHG response, similar to α-SiO_2 (Figure S12b). These results clearly demonstrate that Li_3NbO_4 has no effect on the extremely strong SHG response of NCS $Pb_{1.91}K_{3.22}\square_{0.85}Li_{2.96}Nb_{10}O_{30}$.

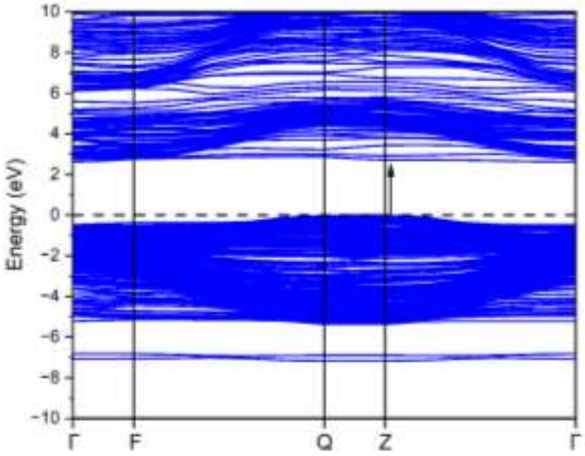

Figure S13. Band structure for $Pb_{1.91}K_{3.22}\square_{0.85}Li_{2.96}Nb_{10}O_{30}$. Black arrow indicates the optical transition from the valence band maximum (VBM) to the conduction band minimum (CBM).

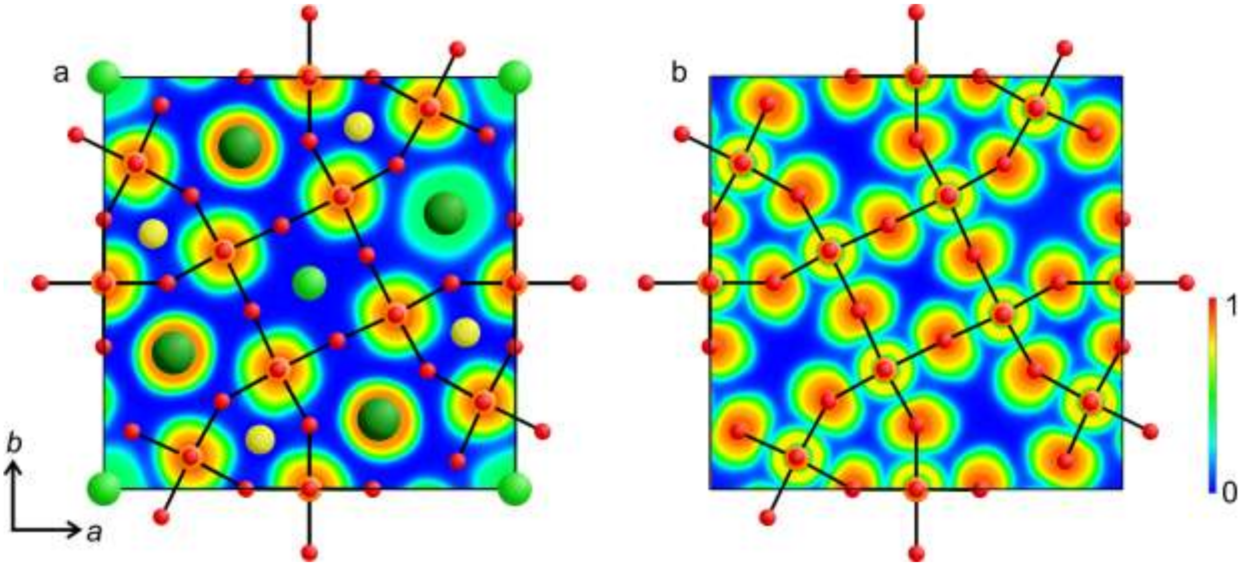

Figure S14. Ball-and-stick models and electron localization function (ELF) diagrams for $Pb_{1.91}K_{3.22}\square_{0.85}Li_{2.96}Nb_{10}O_{30}$ along the *c*-axis with (a) $z = 0$ and (b) $z = 0.5$ (dark green, Pb/K; light green, Pb; yellow, Li; orange, Nb; red, O).

As the crystal structure is disordered, we utilized a supercell code (e.g., *J. Cheminform.* 2016, 8, 17) to obtain a suitable supercell structure for DFT calculations. Based on crystallographic results, the refined occupancy of Li, Pb, and Pb/K was determined to be 0.74(4), 0.5711(12), and 0.8057(13)/0.1943(13), respectively. Accordingly, the resulting structure used in the simulation contains three fully occupied Li, one Pb, and 3K/1Pb in the 3-, 4-, and 5-MRs, respectively, within the unit cell. Although the framework of the distorted NbO_6 octahedra was identical in all 31 configurations with equal Coulomb energy, the configured Pb, K, and Li occupied different positions. Since all configurations used in the simulation were comparable, we used only one result to explain the comprehensive electronic structures.

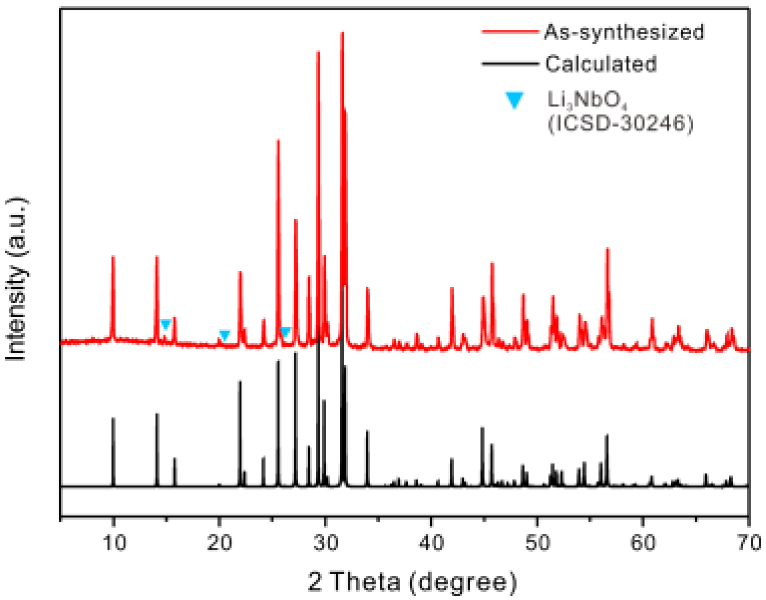

Figure S15. Calculated and experimental PXRD patterns for $Pb_{1.91}K_{3.22}\square_{0.85}Li_{2.96}Nb_{10}O_{30}$.

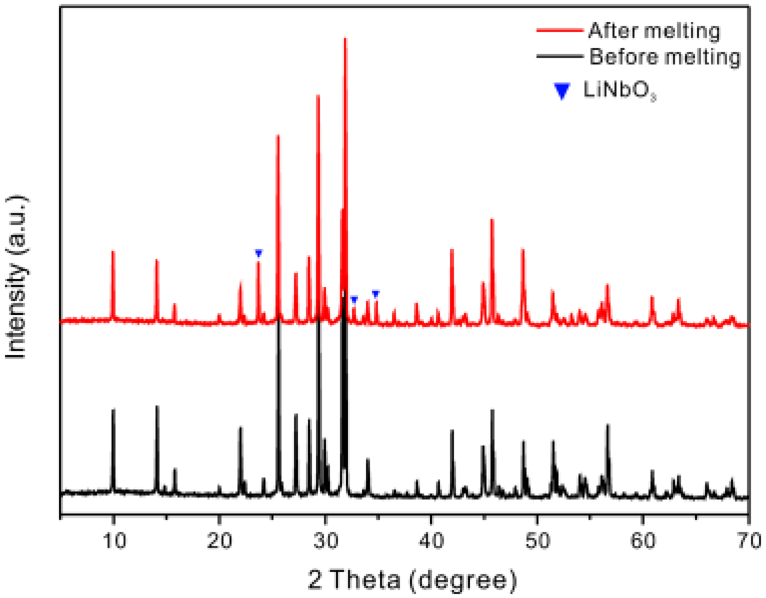

Figure S16. PXRD patterns for $Pb_{1.91}K_{3.22}□_{0.85}Li_{2.96}Nb_{10}O_{30}$ and the melted sample.

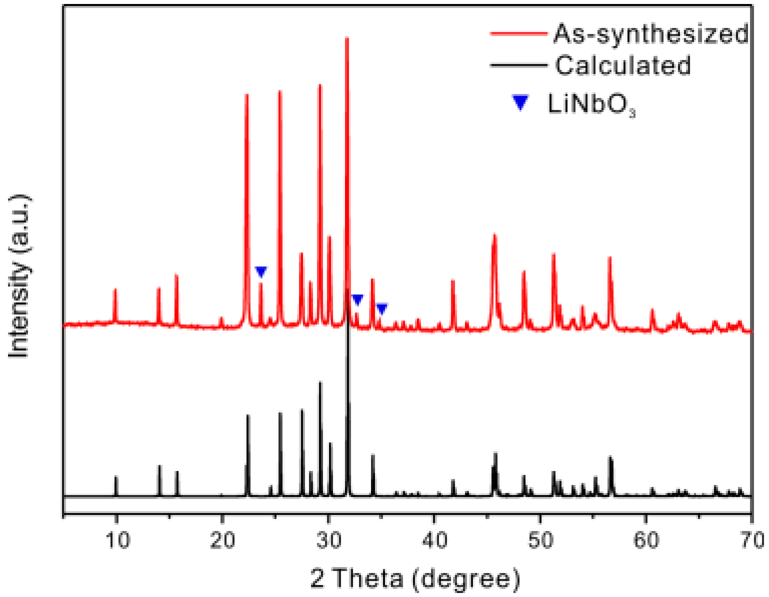

Figure S17. Calculated and experimental PXRD patterns for $Pb_{1.45}K_{3.56}Li_{3.54}Nb_{10}O_{30}$.

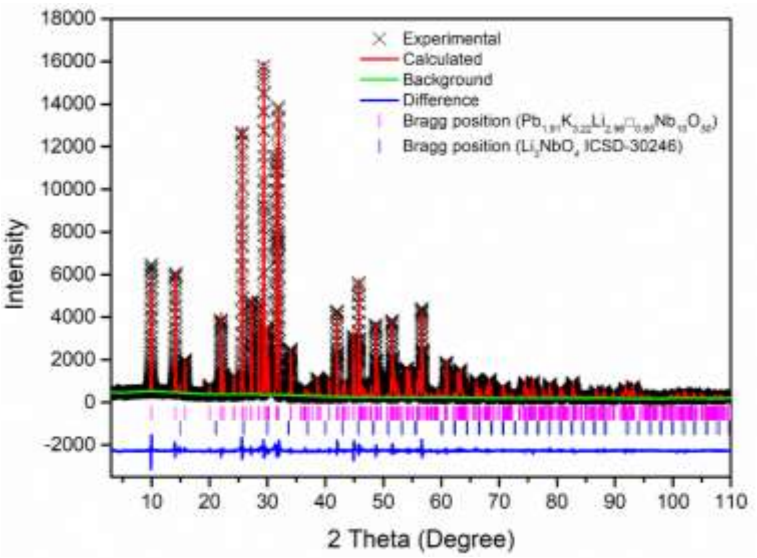

Figure S18. Final Rietveld refinement plot for $Pb_{1.91}K_{3.22}\square_{0.85}Li_{2.96}Nb_{10}O_{30}$.

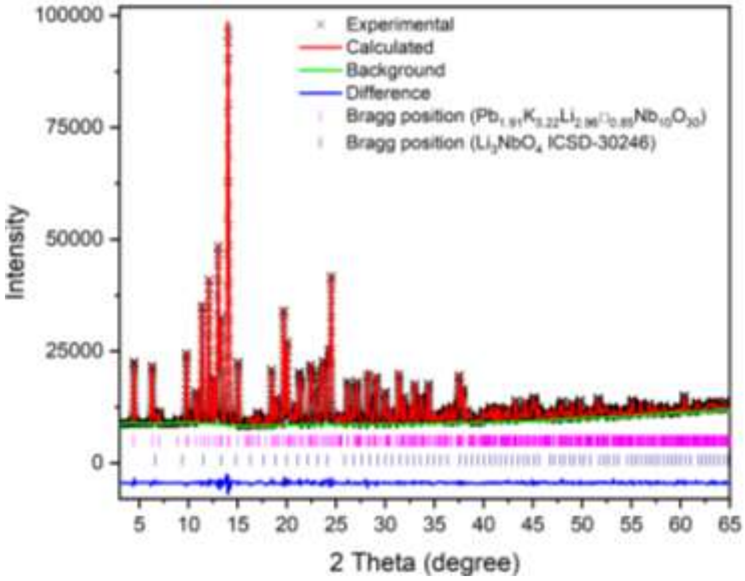

Figure S19. Final Rietveld refinement plot of synchrotron powder X-ray diffraction pattern (λ = 0.68880 Å) for $Pb_{1.91}K_{3.22}\square_{0.85}Li_{2.96}Nb_{10}O_{30}$.

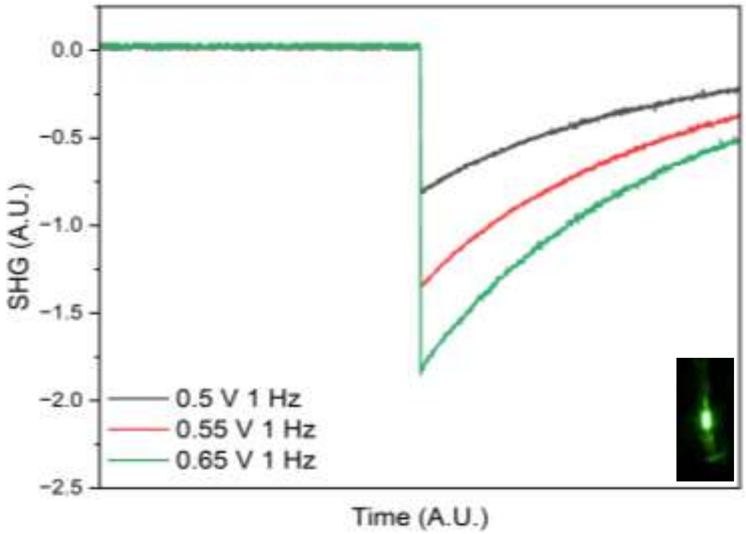

Figure S20. Oscilloscope traces of the SHG signals of $Pb_{1.91}K_{3.22}\square_{0.85}Li_{2.96}Nb_{10}O_{30}$ measured under different laser power.

We performed SHG measurements to observe whether the SHG signal decreases as the material is damaged by the laser. When the power of the pulsed laser was increased and exposed to the sieved sample contained in the capillary, the SHG intensities gradually increased. No damage was observed on the sample surface, and no decrease in SHG signal was found. We also used a pellet and continuous wave (CW) laser for better observation. Even when exposed to pellets while increasing the power of the CW laser to its maximum, no damage was observed on the pellet surface.

References

[1] S. Chu, K. Lin, T. Yang, C. Yu, Y. Cao, Y. Zhang, Y. Sun, Z. Li, X. Jiang, Z. Lin, Q. Li, J. Chen, K. Kato, H. Wu, Q. Huang, X. Xing, *Chem. Commun.* **2020**, *56*, 8384.

[2] K. Lin, P. Gong, S. Chu, Q. Li, Z. Lin, H. Wu, Q. Wang, J. Wang, M. J. Kim, K. Kato, *J. Am. Chem. Soc.* **2020**, *142*, 7480.

[3] K. Lin, P. Gong, J. Sun, H. Ma, Y. Wang, L. You, J. Deng, J. Chen, Z. Lin, K. Kato, H. Wu, Q. Huang, X. Xing, *Inorg. Chem.* **2016**, *55*, 2864.

[4] K. Lin, Z. Zhou, L. Liu, H. Ma, J. Chen, J. Deng, J. Sun, L. You, H. Kasai, K. Kato, M. Takata, X. Xing, *J. Am. Chem. Soc.* **2015**, *137*, 13468.

[5] E. O. Chi, A. Gandini, K. M. Ok, L. Zhang, P. S. Halasyamani, *Chem. Mater.* **2004**, *16*, 3616.

[6] H. Liu, H. Wu, Z. Hu, J. Wang, Y. Wu, H. Yu, *Chem. Mater.* **2022**, *34*, 3501.

[7] C. Wu, X. Jiang, L. Lin, Z. Lin, Z. Huang, M. G. Humphrey, C. Zhang, *Chem. Mater.* **2020**, *32*, 6906.

[8] H. Y. Chang, S. W. Kim, P. S. Halasyamani, *Chem. Mater.* **2010**, *22*, 3241.

[9] H. Yu, N. Z. Koocher, J. M. Rondinelli, P. S. Halasyamani, *Angew. Chem. Int. Ed.* **2018**, *57*, 6100.

[10] M. Luo, Y. Song, F. Liang, N. Ye, Z. Lin, *Inorg. Chem. Front.* **2018**, *5*, 916.

[11] G. Zou, C. Lin, H. Jo, G. Nam, T.-S. You, K. M. Ok, *Angew. Chem. Int. Ed.* **2016**, *55*, 12078.

[12] K. Lin, H. Wu, F. Wang, Y. Rong, J. Chen, J. Deng, R. Yu, L. Fang, Q. Huang, X. Xing, *Dalton Trans.* **2014**, *43*, 7037.

[13] N. Yabuuchi, M. Takeuchi, M. Nakayama, H. Shiiba, M. Ogawa, K. Nakayama, T. Ohta, D. Endo, T. Ozaki, T. Inamasu, K. Sato, S. Komaba, *PNAS* **2015**, *112*, 7650.
