# Supplementary material for: A Polar Tetragonal Tungsten Bronze with Colossal Second‐Harmonic Generation
Source: Adv Sci (Weinh). 2023 Apr 23;10(19):2301374. doi: 10.1002/advs.202301374 (PMC10323606; doi:10.1002/advs.202301374)

checkCIF/PLATON report

Structure factors have been supplied for datablock(s) TTBCS, TTBNCS

THIS REPORT IS FOR GUIDANCE ONLY. IF USED AS PART OF A REVIEW PROCEDURE FOR PUBLICATION, IT SHOULD NOT REPLACE THE EXPERTISE OF AN EXPERIENCED CRYSTALLOGRAPHIC REFEREE.

No syntax errors found. CIF dictionary Interpreting this report

Datablock: TTBNCS

Bond precision: Nb- O = 0.0124 A Wavelength=0.71073

Cell:	a=12.533(5)	b=12.533(5)	c=4.016(2)
	alpha=90	beta=90	gamma=90
Temperature:	297 K		

	Calculated	Reported
Volume	630.8(6)	630.9(6)
Space group	P 4 b m	P 4 b m
Hall group	P 4 -2ab	P 4 -2ab
Moiety formula	Nb10 O30 Pb1.91, 3.216(K), 2.962(Li)	Nb10 O30 Pb1.91, 3.22(K), 2.962(Li)
Sum formula	K3.22 Li2.96 Nb10 O30 Pb1.91	K3.22 Li2.96 Nb10 O30 Pb1.91
Mr	1951.99	1951.28
Dx,g cm-3	5.139	5.136
Z	1	1
Mu (mm-1)	17.732	17.705
F000	876.9	877.0
F000'	850.57	
h,k,lmax	16,16,5	16,16,5
Nref	852[484]	846
Tmin,Tmax	0.081,0.256	0.382,0.746
Tmin'	0.010	

Correction method= # Reported T Limits: Tmin=0.382 Tmax=0.746
AbsCorr = MULTI-SCAN

Data completeness= 1.75/0.99 Theta(max)= 28.421

R(reflections)= 0.0268(846) wR2(reflections)= 0.0619(846)

S = 1.204 Npar= 47

The following ALERTS were generated. Each ALERT has the format
test-name_ALERT_alert-type_alert-level.
Click on the hyperlinks for more details of the test.

Alert level C

```
STRVA01_ALERT_4_C          Flack test results are ambiguous.
           From the CIF: _refine_ls_abs_structure_Flack    0.490
           From the CIF: _refine_ls_abs_structure_Flack_su    0.030
PLAT042_ALERT_1_C Calc. and Reported MoietyFormula Strings  Differ     Please Check
PLAT077_ALERT_4_C Unitcell Contains Non-integer Number of Atoms ..     Please Check
PLAT202_ALERT_3_C Isotropic non-H Atoms in Anion/Solvent .........          1 Check
              Li1
PLAT220_ALERT_2_C NonSolvent   Resd 1  O   Ueq(max)/Ueq(min) Range        5.7 Ratio
PLAT220_ALERT_2_C NonSolvent   Resd 1 Pb   Ueq(max)/Ueq(min) Range        5.0 Ratio
PLAT242_ALERT_2_C Low    'MainMol' Ueq as Compared to Neighbors of        Nb2 Check
PLAT971_ALERT_2_C Check Calcd Resid. Dens.  0.60Ang From O3              1.68 eA-3
PLAT972_ALERT_2_C Check Calcd Resid. Dens.  1.38Ang From O5             -1.67 eA-3
PLAT975_ALERT_2_C Check Calcd Resid. Dens.  0.47Ang From O5      .       0.89 eA-3
PLAT975_ALERT_2_C Check Calcd Resid. Dens.  0.53Ang From O1      .       0.88 eA-3
PLAT975_ALERT_2_C Check Calcd Resid. Dens.  0.60Ang From O1      .       0.86 eA-3
PLAT975_ALERT_2_C Check Calcd Resid. Dens.  1.08Ang From O1      .       0.85 eA-3
PLAT975_ALERT_2_C Check Calcd Resid. Dens.  0.64Ang From O4      .       0.70 eA-3
PLAT975_ALERT_2_C Check Calcd Resid. Dens.  0.53Ang From O2      .       0.66 eA-3
PLAT975_ALERT_2_C Check Calcd Resid. Dens.  0.44Ang From O2      .       0.61 eA-3
PLAT975_ALERT_2_C Check Calcd Resid. Dens.  0.57Ang From O4      .       0.55 eA-3
PLAT975_ALERT_2_C Check Calcd Resid. Dens.  0.95Ang From O2      .       0.53 eA-3
```

Alert level G

```
PLAT004_ALERT_5_G Polymeric Structure Found with Maximum Dimension          3 Info
PLAT083_ALERT_2_G SHELXL Second Parameter in WGHT  Unusually Large       9.92 Why ?
PLAT111_ALERT_2_G ADDSYM Detects New (Pseudo) Centre of Symmetry .        100 %Fit
PLAT113_ALERT_2_G ADDSYM Suggests Possible Pseudo/New  Space Group     P4/mbm Check
              Check Model Parameter Symmmetry for Reflection Data Support
PLAT168_ALERT_4_G The CIF-Embedded .res File Contains EXYZ Records          1 Report
PLAT171_ALERT_4_G The CIF-Embedded .res File Contains EADP Records          1 Report
PLAT300_ALERT_4_G Atom Site Occupancy of Li1        Constrained at     0.7406 Check
PLAT301_ALERT_3_G Main Residue  Disorder .............(Resd  1  )         12% Note
PLAT302_ALERT_4_G Anion/Solvent/Minor-Residue Disorder (Resd  2  )       100% Note
PLAT302_ALERT_4_G Anion/Solvent/Minor-Residue Disorder (Resd  3  )       100% Note
PLAT910_ALERT_3_G Missing # of FCF Reflection(s) Below Theta(Min).          1 Note
PLAT913_ALERT_3_G Missing # of Very Strong Reflections in FCF ....          1 Note
PLAT961_ALERT_5_G Dataset Contains no Negative Intensities .......     Please Check
```

---

0 **ALERT level A** = Most likely a serious problem - resolve or explain
0 **ALERT level B** = A potentially serious problem, consider carefully
18 **ALERT level C** = Check. Ensure it is not caused by an omission or oversight
13 **ALERT level G** = General information/check it is not something unexpected

```
 1 ALERT type 1 CIF construction/syntax error, inconsistent or missing data
17 ALERT type 2 Indicator that the structure model may be wrong or deficient
 4 ALERT type 3 Indicator that the structure quality may be low
 7 ALERT type 4 Improvement, methodology, query or suggestion
 2 ALERT type 5 Informative message, check
```

Datablock: TTBCS

Bond precision: Nb- O = 0.0036 A Wavelength=0.71073

Cell:	a=12.5893(5)	b=12.5893(5)	c=3.9612(2)
	alpha=90	beta=90	gamma=90
Temperature:	296 K		

	Calculated	Reported
Volume	627.81(6)	627.81(6)
Space group	P 4/m b m	P 4/m b m
Hall group	-P 4 2ab	-P 4 2ab
Moiety formula	Nb10 O30 Pb1.45, 3.56(K), 3.546(Li)	Nb10 O30 Pb1.45, 3.56(K), 3.546(Li)
Sum formula	K3.56 Li3.55 Nb10 O30 Pb1.45	K3.56 Li3.55 Nb10 O30 Pb1.45
Mr	1872.93	1872.93
Dx,g cm-3	4.954	4.953
Z	1	1
Mu (mm-1)	14.764	14.752
F000	847.0	847.0
F000’	822.20	
h,k,lmax	16,16,5	16,16,5
Nref	477	476
Tmin,Tmax	0.176,0.444	0.472,0.746
Tmin’	0.125	

Correction method= # Reported T Limits: Tmin=0.472 Tmax=0.746
AbsCorr = MULTI-SCAN

Data completeness= 0.998 Theta(max)= 28.400

R(reflections)= 0.0329(472) wR2(reflections)= 0.0653(476)

S = 1.081 Npar= 50

The following ALERTS were generated. Each ALERT has the format
test-name_ALERT_alert-type_alert-level.
Click on the hyperlinks for more details of the test.

Alert level C

```
PLAT077_ALERT_4_C Unitcell Contains Non-integer Number of Atoms ..     Please Check
PLAT088_ALERT_3_C Poor Data / Parameter Ratio ....................       9.52 Note
PLAT241_ALERT_2_C High   'MainMol' Ueq as Compared to Neighbors of         O3 Check
PLAT242_ALERT_2_C Low    'MainMol' Ueq as Compared to Neighbors of        Nb2 Check
PLAT242_ALERT_2_C Low    'MainMol' Ueq as Compared to Neighbors of         O2 Check
PLAT313_ALERT_2_C Oxygen with Three Covalent Bonds (rare) ........         O2 Check
PLAT313_ALERT_2_C Oxygen with Three Covalent Bonds (rare) ........         O3 Check
PLAT313_ALERT_2_C Oxygen with Three Covalent Bonds (rare) ........         O5 Check
PLAT906_ALERT_3_C Large K Value in the Analysis of Variance ......      2.065 Check
PLAT971_ALERT_2_C Check Calcd Resid. Dens.  0.87Ang From Pb2             1.74 eA-3
PLAT972_ALERT_2_C Check Calcd Resid. Dens.  0.57Ang From Pb2B           -1.62 eA-3
PLAT975_ALERT_2_C Check Calcd Resid. Dens.  1.03Ang From O4      .       0.73 eA-3
PLAT975_ALERT_2_C Check Calcd Resid. Dens.  0.80Ang From O1      .       0.66 eA-3
PLAT975_ALERT_2_C Check Calcd Resid. Dens.  0.71Ang From O4      .       0.47 eA-3
PLAT976_ALERT_2_C Check Calcd Resid. Dens.  1.06Ang From O2      .      -0.90 eA-3
```

Alert level G

```
PLAT004_ALERT_5_G Polymeric Structure Found with Maximum Dimension          3 Info
PLAT083_ALERT_2_G SHELXL Second Parameter in WGHT  Unusually Large      15.31 Why ?
PLAT168_ALERT_4_G The CIF-Embedded .res File Contains EXYZ Records          1 Report
PLAT171_ALERT_4_G The CIF-Embedded .res File Contains EADP Records          2 Report
PLAT300_ALERT_4_G Atom Site Occupancy of Li1       Constrained at      0.8865 Check
PLAT301_ALERT_3_G Main Residue  Disorder .............(Resd  1  )         15% Note
PLAT302_ALERT_4_G Anion/Solvent/Minor-Residue Disorder (Resd  2  )       100% Note
PLAT302_ALERT_4_G Anion/Solvent/Minor-Residue Disorder (Resd  3  )       100% Note
PLAT910_ALERT_3_G Missing # of FCF Reflection(s) Below Theta(Min).          1 Note
PLAT933_ALERT_2_G Number of HKL-OMIT Records in Embedded .res File          1 Note
PLAT961_ALERT_5_G Dataset Contains no Negative Intensities .......     Please Check
```

---

0 **ALERT level A** = Most likely a serious problem - resolve or explain
0 **ALERT level B** = A potentially serious problem, consider carefully
15 **ALERT level C** = Check. Ensure it is not caused by an omission or oversight
11 **ALERT level G** = General information/check it is not something unexpected

0 ALERT type 1 CIF construction/syntax error, inconsistent or missing data
14 ALERT type 2 Indicator that the structure model may be wrong or deficient
4 ALERT type 3 Indicator that the structure quality may be low
6 ALERT type 4 Improvement, methodology, query or suggestion
2 ALERT type 5 Informative message, check

It is advisable to attempt to resolve as many as possible of the alerts in all categories. Often the minor alerts point to easily fixed oversights, errors and omissions in your CIF or refinement strategy, so attention to these fine details can be worthwhile. In order to resolve some of the more serious problems it may be necessary to carry out additional measurements or structure refinements. However, the purpose of your study may justify the reported deviations and the more serious of these should normally be commented upon in the discussion or experimental section of a paper or in the "special_details" fields of the CIF. checkCIF was carefully designed to identify outliers and unusual parameters, but every test has its limitations and alerts that are not important in a particular case may appear. Conversely, the absence of alerts does not guarantee there are no aspects of the results needing attention. It is up to the individual to critically assess their own results and, if necessary, seek expert advice.

Publication of your CIF in IUCr journals

A basic structural check has been run on your CIF. These basic checks will be run on all CIFs submitted for publication in IUCr journals (*Acta Crystallographica*, *Journal of Applied Crystallography*, *Journal of Synchrotron Radiation*); however, if you intend to submit to *Acta Crystallographica Section C* or *E* or *IUCrData*, you should make sure that full publication checks are run on the final version of your CIF prior to submission.

Publication of your CIF in other journals

Please refer to the *Notes for Authors* of the relevant journal for any special instructions relating to CIF submission.

PLATON version of 28/11/2022; check.def file version of 28/11/2022

Datablock TTBNCS - ellipsoid plot

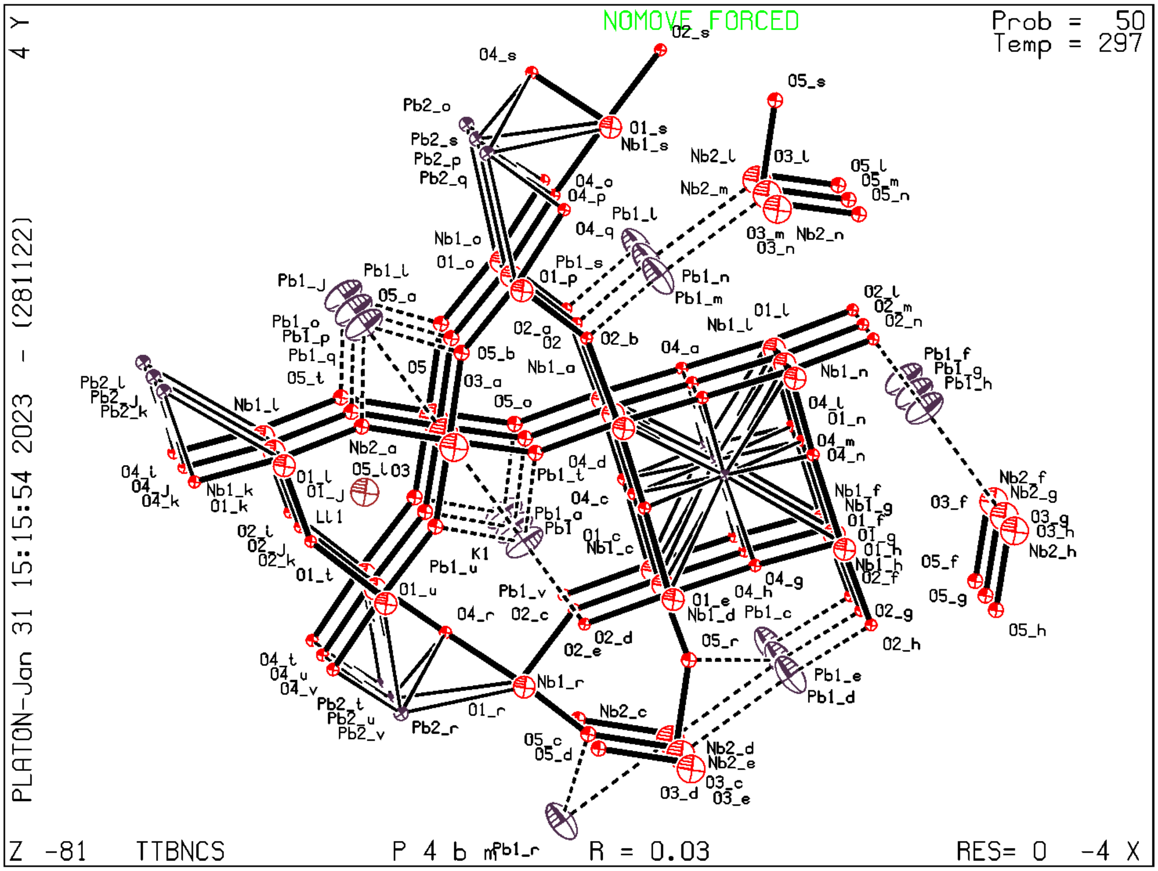

Datablock TTBCS - ellipsoid plot

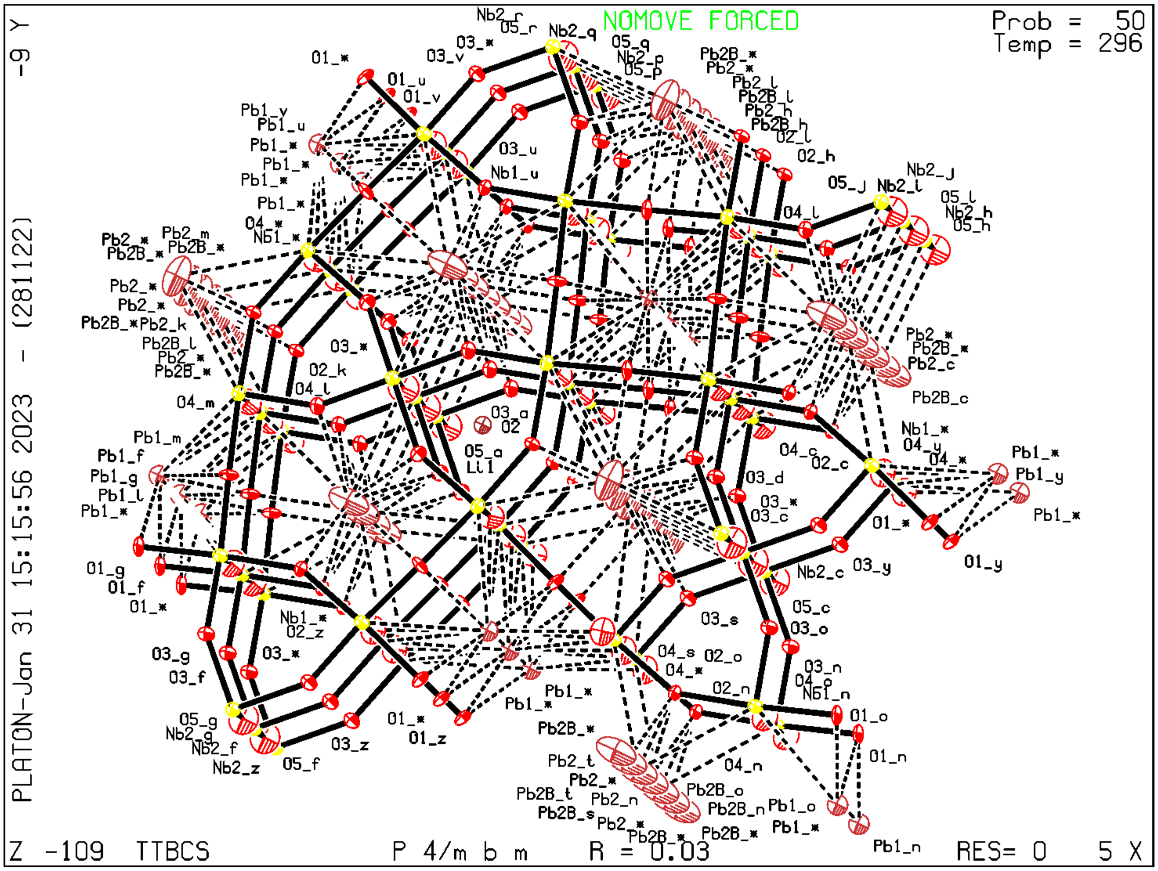

Supplement: Supplementary file 2 — Supporting Information [file ADVS-10-2301374-s002.zip › CHECKCIF.pdf]
